# Reconstructing the Evolutionary History of *Pinna nobilis*: New Genetic Signals from the Past of a Species on the Brink of Extinction

**DOI:** 10.3390/ani14010114

**Published:** 2023-12-28

**Authors:** Daria Sanna, Ilenia Azzena, Chiara Locci, Pavel Ankon, Petar Kružić, Chiara Manfrin, Alberto Pallavicini, Saul Ciriaco, Marco Segarich, Edoardo Batistini, Fabio Scarpa, Marco Casu

**Affiliations:** 1 Department of Biomedical Sciences, University of Sassari, Viale San Pietro 43b, 07100 Sassari, Italy; iazzena@uniss.it (I.A.); c.locci3@phd.uniss.it (C.L.); fscarpa@uniss.it (F.S.); 2 Department of Veterinary Medicine, University of Sassari, Via Vienna 2, 07100 Sassari, Italy; marcasu@uniss.it; 3 Department of Biology, Faculty of Science, University of Zagreb, Horvatovac 102a, 10000 Zagreb, Croatia; pavel.ankon@gmail.com (P.A.); petar.kruzic@biol.pmf.hr (P.K.); 4 Department of Life Sciences, University of Trieste, Via L. Giorgieri 5, 34127 Trieste, Italy; cmanfrin@units.it (C.M.); pallavic@units.it (A.P.); 5 WWF AMP Miramare, Via Beirut 2/4, 34151 Trieste, Italy; saul@ampmiramare.it; 6 Shoreline Soc. Coop., AREA Science Park, Padriciano 99, 34149 Trieste, Italy; marco.segarich@shoreline.it (M.S.); edoardo.batistini@shoreline.it (E.B.)

**Keywords:** fan mussel, mtDNA, cytochrome c oxidase subunit I, evolution, molecular dating

## Abstract

**Simple Summary:**

*Pinna nobilis*, a species of marine shellfish living in the Mediterranean Sea, is at a high risk of extinction due to a not-entirely-known disease that started affecting its populations in 2016. In this paper, we reported the main traits of its evolutionary history to understand how this species evolved over time and space from the moment its ancestor entered the Mediterranean. To achieve this goal, we analysed a total of 469 sequences from all over the Mediterranean Sea. Our research showed that *P. nobilis* evolved from its ancestor about 2.5 million years ago, following a rapid and catastrophic entry of waters from the Atlantic Ocean that pushed the *P. nobilis* ancestor into the Mediterranean around 5.3 million years ago. Our results also suggest that the central part of the western Mediterranean was the first marine area where this species settled and, later on, it spread to the Adriatic and the eastern part of the basin. This information is of twofold importance, as it helps us to understand how this species adapted to the Mediterranean over time and may be the basis of present and future restocking plans which want to take into consideration the reconstruction of pre-existing genetic variability.

**Abstract:**

*Pinna nobilis*, commonly known as the noble pen shell, is a marine bivalve endemic to the Mediterranean Sea. Unfortunately, due to a multifactorial disease that began affecting its populations in 2016, the species is currently facing the threat of extinction. To gain insights into the evolutionary history of *P. nobilis* before the mass mortality event (MME), and to obtain a comprehensive understanding of how evolutionary processes led to the adaptation of the species into the Mediterranean Sea, phylogenetic and phylogeographic analyses were carried out. The dataset analysed includes 469 sequences of COI gene fragment both from GenBank and the present study (100). The analysis performed evidenced that *P. nobilis* diverged about 2.5 mya, after the entrance of its ancestor into the Mediterranean Sea following the Zanclean flood (5.33 mya). Moreover, our results suggest that the starting point of colonisation was the central part of the western Mediterranean basin, with the eastern basin being populated subsequently. From a conservational viewpoint, these results provide important hints for present and future restocking plans, helping to reconstruct the pre-existing genetic variability in sites where the species became extinct.

## 1. Introduction

*Pinna nobilis* Linnaeus, 1758, commonly known as fan mussel or noble pen shell, is a long-lived, large species of marine bivalve endemic to the Mediterranean Sea and belonging to the family Pinnidae (Mollusca: Bivalvia) [1]. Over the past few decades, the taxonomy of the family Pinnidae Leach, 1819, has undergone several revisions. To date, two still-living genera belong to this taxon: *Pinna* Linnaeus, 1758, and *Atrina* Gray, 1847. Indeed, Lemer et al. [2] made the most recent change to the taxonomic status within this family, proposing a new status for the genus *Streptopinna* E. von Martens, 1880, and now considering it as a subgenus (status nov.) of *Pinna*.

Due to its morphological and ecological characteristics, *P. nobilis* is a pteriomorphian bivalve (infraclass: Pteriomorphia) inhabiting the Mediterranean Sea since the Miocene era [3]. This filter-feeding species is commonly found on the coastal sandy sediment of the infralittoral, from 0.5 to 60 m, often in *Posidonia oceanica* meadows, where it lives semiburied, anchoring to the substratum thanks to its byssus threads, which glue to pebbles, sand, small fragments of robust biodetritic material, as well as the roots and rhizomes of *P. oceanica* [4].

*Pinna nobilis* has been exploited by humans for various purposes over the centuries [1,5,6,7,8,9]. It is worth noting that, in southern Italy, particularly in Apulia (Taranto), and in the Sardinia Island (Sant’Antioco), a longstanding tradition of harvesting the byssus produced by *P. nobilis* occurred, with the aim of creating luxurious fabrics and textiles. Lastly, populations of *P. nobilis* were severely impacted by indirect human activities, including boat anchoring, pollution, and habitat fragmentation [10,11].

Because of all these direct and indirect activities having impacted the species over time and space, *P. nobilis* experienced a strong demographic decline in all of its range, which accelerated significantly by the late 1980s [12]. To invert this trend, at the beginning of the 1990s, *P. nobilis* was included in a full protection regime under Annex IV of the EU Habitats Directive (European Council Directive 92/43/EEC) and Annex II of the Barcelona Convention (SPA/BD Protocol 1995). Moreover, many countries have enacted their own legislative measures, establishing conservation protocols to address the historical exploitation and continuous threats facing *P. nobilis* (e.g., in Italy, Marine Strategy Monitoring Program, Article 11 of Legislative Decree 190/210; in Slovenia, Annexes 1 and 2 of the Regulations for the protection of wild flora and fauna, Official Gazette of the Republic of Slovenia, nos. 46/04, 109/04, 84/05, 115/07, and 32/08; in Croatia, Croatian Nature Protection Act, Official Gazette 144/2013, 73/2016).

In a few decades, the great commitment of both the European community and single countries to protect the species led to a remarkable revival of its populations ([13] and references therein). This occurrence was, e.g., testified during the sampling activities done for several scientific studies on *P. nobilis* [5,14,15,16,17,18,19,20,21,22,23,24,25,26,27,28].

Unfortunately, an abnormal mortality of *P. nobilis* started in 2016, initially involving the populations located in the centre and southernmost coasts of Spain, together with the Balearic Islands [29], with death rates reaching up to 100%.

Since this first warning in southern Spain, countless mass mortality events (MMEs) have gradually been reported in an eastward direction in the western Mediterranean basin, involving northern Spain, France, and Italy, further reaching the central and the eastern part of the Mediterranean (Tunisia, Greece, and Turkey), and lastly affecting the Adriatic Sea, e.g., Slovenia, Croatia, Bosnia, and Herzegovina [29,30,31,32,33,34,35,36,37,38,39]. The initial investigations into the causes of the MME of *P. nobilis* primarily concentrated on the search for protozoa [29,30,32,33,40,41,42], and histological analysis on the first affected individuals of *P. nobilis* revealed the presence of a haplosporidian-like parasite (*Haplosporidium pinnae*) within the digestive gland [29,30,40], initially believed to be host-specific for fan mussels. Subsequent studies identified several bacterial species as potential pathogens contributing to the MME of *P. nobilis* [13,35,36,37,39,43,44,45,46], indicating that the disease is a multifactorial pathology [13,47,48]. Scarpa et al. [13] also reported the finding of *H. pinnae* in other bivalve species collected before 2016. These results highlighted that *H. pinnae* is not species-specific as previously hypothesised, and that this protozoan was present in the Mediterranean Sea even before the start of the *P. nobilis* MME.

Only a few populations of *P. nobilis* survived the MME based on the most recent publications; however, we are aware that some populations disappear within a few months (e.g., Croatian populations, Čižmek et al. [35]) [49]. These populations are primarily situated in estuaries and isolated coastal lagoons in France, Italy, Spain [50], Greece [51], and the Sea of Marmara [52]. Notably, even if there are no recent surveys concerning the status of *P. nobilis* along the Tunisian coastlines, some unpublished studies indicate thriving populations in the Kerkennah Islands, Monastir Bay, and Bizerte Lagoon [53]. Even if the specific reasons for why these populations have remained unaffected are not yet understood, differences in salinity and temperature, in comparison to open waters, could play a role in their survival [34,50,53,54].

As a result of the MME, the conservation status of *P. nobilis* was reassessed, leading to its classification being updated from endangered to critically endangered [55]. In response to this critical situation, several Mediterranean organisations launched ex situ conservation programs, with an emphasis on captive breeding and reintroduction initiatives [45,55,56], and there is a strong emphasis on safeguarding the remaining, albeit few, wild populations of *P. nobilis* in the Mediterranean Sea [56]. Against this backdrop, the European community funded two ongoing LIFE projects devoted to the conservation and restocking of fan mussels: LIFE PINNARCA (https://www.lifepinnarca.com/the-project/) and LIFE PINNA (https://www.lifepinna.eu/en/the-project/). The second project involves the relocation of individuals from donor areas (sited in the northern Adriatic Sea) to receiving areas (in some sites of the western Mediterranean). Among these actions, there is an in-depth study of the genetic structure of *P. nobilis* to restore populations with genetic backgrounds like that which characterised extinct populations.

In such a context, several molecular studies have been performed on the genetic variability of *P. nobilis* so far [14,22,25,49,51,57,58,59,60,61]. The primary objective of the majority of these papers was to infer the genetic variability of *P. nobilis* after the protection plans started in the 1990s. Among them, Sanna et al. [14] provided a comprehensive Mediterranean-scale assessment of the genetic variability of *P. nobilis* using mitochondrial markers (cytochrome c oxidase subunit I (COI) and 16S ribosomal subunit genes) and combining their sequences with those obtained in previous studies [22,25]. Results highlighted high levels of genetic variability across the following marine ecoregions: (1) the western Mediterranean and the Ionian Sea; (2) the Adriatic Sea; (3) the Aegean Sea and Tunisian coastal regions. Furthermore, authors set the genetic boundary between the western and eastern Mediterranean basins in the Ionian Sea, thus suggesting that, for *P. nobilis*, the Sicilian straits do not represent a boundary for larval dispersal. These results were then corroborated by Sarafidou et al. [51], who analysed the residual genetic variation in the surviving populations after the MME from sites in the Aegean and Ionian Seas. Furthermore, based on the COI analyses, and consistent with what was previously hypothesised by Sanna et al. [14], Wesselman et al. [58] suggested that *P. nobilis* is characterised by a single mitochondrial haplogroup that experienced a recent population expansion starting from a small, original population. González-Wangüemert et al. [59] utilised microsatellite markers [62] to analyse different populations of *P. nobilis* sampled between 2010 and 2011 along the Spanish Mediterranean coast. Results showed a high genetic diversity and significant differentiation among postlarvae samples, but not among adult populations, suggesting that the overall genetic connectivity retrieved was correlated to both marine currents and dispersal models.

In this context, our research group had the unique opportunity to analyse “new” samples of *P. nobilis* collected before the mass mortality began. The possibility of genotyping them, considering that the species is on the brink of extinction and some restoration programs are based on the possibility of translocating individuals from the few remaining refuge areas to other areas where the species is now disappeared, has a relevant conservation importance. Indeed, past genetic variation patterns should be preserved when possible, and phylogeography studies have demonstrated the capacity to help conceive and address conservation measures (see, e.g., [63,64,65]).

In light of such a background, the present study aimed to analyse the largest dataset of mitochondrial sequences available to understand the evolutionary patterns of genetic variability in the Mediterranean for *P. nobilis*. Analyses were performed using all data from populations that were sampled prior to the MME, and many of these populations are now becoming extinct. Utilising samples from populations not yet impacted by the MME, we investigated the genetic variation patterns without the influence of evolutionary forces resulting from the severe population collapse observed in *P. nobilis*. The combined effects of genetic drift, including bottleneck and/or founder effects, natural selection, and selective sweep, may have led to the disappearance of informative haplotypes or the amplification of previously uncommon ones. Our goal was to create a detailed portrayal of the historical genetic variability of *P. nobilis*, aiming to comprehend its potential survival in the face of mass mortality. We sought to understand how evolutionary forces might impact the species in the future.

For these reasons, our genetic analyses relied on the mitochondrial marker for which sequences from all the populations investigated to date are available in GenBank, and over a hundred newly obtained sequences from individuals collected before the MME in previously unexplored areas. Mitochondrial DNA (mtDNA) served as a valuable molecular marker for inferring population dynamics, dispersal patterns, and evolutionary history across various species [66].

In summary, the study pursued two main objectives: firstly, to depict the phylogeographic patterns and genetic variation in *P. nobilis* before the MME, aiding the development of strategies for the recovery and revival of populations resembling those that became extinct. Secondly, to validate the hypothesis proposed by Sanna et al. [14], establishing the genetic boundary between the western and eastern Mediterranean for this species eastward of the Sicilian strait through analyses of three new Adriatic populations.

A further aim of the present study was to shed light on the temporal and geographical origin of the species *P. nobilis*, whose unique Mediterranean fossil records were found in a late Pliocene–early Pleistocene site in northern Italy [67]. In this context, the Mediterranean Sea gradually refilled with seawater from the Atlantic Ocean after the Messinian salinity crisis ended, thus opening the door for the rise of new marine species in the re-emerging sea (references in Garcia-Castellanos [68]). Many of these new species were descended from Mediterranean ancestors that survived the desiccation, while other species migrated from the Atlantic Ocean. In the aftermath of the Messinian salinity crisis, the interplay between surviving Mediterranean species and Atlantic colonisers resulted in the emergence of numerous endemic species. In such a remarkable biodiversity mosaic, it would be important to gain insights into the evolutionary patterns of *P. nobilis* which allowed its diffusion in the Mediterranean. Indeed, based on these data, we can provide hints to formulate more informed predictions regarding the species’ potential for recovery after the population decline caused by the MME.

## 2. Materials and Methods

### 2.1. Sample Collection

The dataset analysed in the present study included all the sequences of *Pinna nobilis* collected before the MME. They were either taken from GenBank (n = 369) [14,22,25,58] or obtained in the present study (n = 100) from the fresh tissues of samples collected in the past few years (see Table 1 for details).

Notably, the sequences from the Gulf of Trieste (the north of the Adriatic Sea) used in the present study belonged to populations sampled in 2018 when the northern Adriatic was not yet reached by the MME. For the collection of tissues from still-living individuals, we used a specific nonlethal sampling method, performed by scuba divers, which was developed by these authors [14] and which did not cause significant damage to the shells and soft tissues of *P. nobilis*. With this method, and with the recently received approval of the Italian “Istituto Superiore per la Protezione e la Ricerca Ambientale (ISPRA)” and “Ministero dell’Ambiente e della Tutela del Territorio e del Mare” [13], small fragments of mantle tissue from individuals with a minimally invasive technique were taken. No field studies involving the manipulation, dislocation, or removal of *P. nobilis* individuals were performed. For each location under protection, all necessary permits were obtained for the sampling activities by the authority responsible for each protected area. In Italy, the collection of samples from the Gulf of Trieste (Miramare) was performed according to a waiver to the Presidential Decree 357/97, proposed by the Scientific Directorate of the Marine Protected Area of Miramare (Trieste) (Prot. MATM PNM 0028355 of 10/10/2019). In Croatia, the collection of *P. nobilis* specimens within the national parks of Mljlet and Telašćica was performed under a sampling licence issued by the Ministry of Environmental Protection and Energy (KLASA: UP/I-612-07/16-48/103, URBROJ: 517-07-1-1-1-16-4).

**Figure 1:**
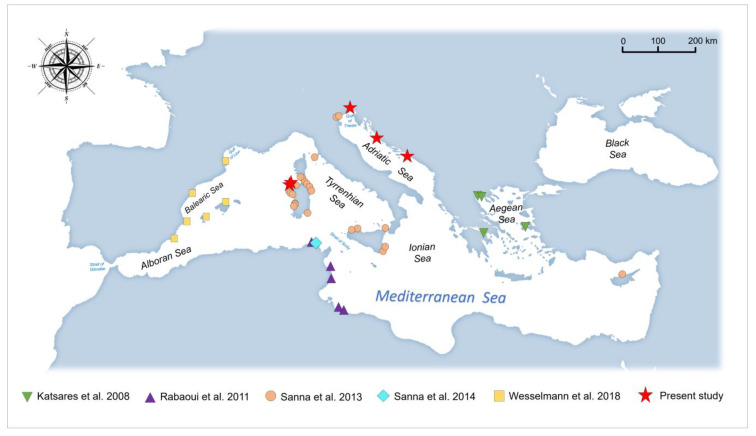
Map of the sampling sites. The map shows the geographical locations for the sequences obtained in the present study along with those from previous research [14,22,25,57,58].

Overall, the dataset analysed included a total of 469 sequences, 100 of which were newly obtained for the present study (representing 21% of the whole analysed dataset) and belonging to areas sited in the following 11 Mediterranean biogeographic sectors according to Bianchi et al. [69] (Table 1 and Figure 1): (1) Algerian and north Tunisian coasts; (2) Balearic Sea to Sardinia Sea; (3) Gulf of Lions; (4) southern Tyrrhenian Sea; (5) Straits of Messina; (6) Ionian Sea; (7) northern Adriatic Sea; (8) central Adriatic Sea; (9) northern Aegean Sea; (10) southern Aegean Sea; (11) Levant Sea.

### 2.2. Molecular Analyses

Total genomic DNA was isolated from a portion of mantle tissue using the Macherey-Nagel Nucleo Spin Tissue Kit (MACHEREY-NAGEL GmbH and Co. KG, Düren, Germany) following the supplier’s instructions. DNA solutions were quantified using the Nanodrop™ Lite Spectrophotometer (by Thermo Scientific; Waltham, MA, USA), which showed an average yield of 54 ng/µL. A portion of the mitochondrial cytochrome c oxidase sub. I gene (COI) was amplified by standard PCR with specific primers for COI (L: 5′-GGTTGAACTATHTATCCNCC-3′ and H: 5′-GAAATCATYCCAAAAGC-3′) designed by the authors [14], which allowed us to obtain a COI fragment that was 338 base pairs long. Reactions were carried out in a total volume of 25 µL. On average, 10 ng of total genomic DNA were combined with 0.6 µM of each primer and one pellet of PuReTaq Ready-To-Go PCR beads (GE Healthcare, Wauwatosa, WI, USA) containing stabilizers, 4 ng of bovine serum albumin (BSA), deoxynucleotide triphosphates, 2.5 units of PuReTaq DNA polymerase, and reaction buffer. When a bead was reconstituted to a 25 µL final volume, the concentration of each dNTP and MgCl_2_ resulted in 200 µM and 1.5 mM, respectively. PCRs were performed in a GeneAmp PCR System 9700 Thermal Cycler (Applied Biosystems, Waltham, MA, USA), programmed as follows: 1 cycle of 4 min at 94 °C, 35 cycles of 30 s at 94 °C, 30 s at 46 °C, and 30 s at 72 °C. At the end, a post-treatment of 10 min at 72 °C and a final cooling at 4 °C were carried out. Both positive (high-quality DNA samples from the same species) and negative controls were used to test the effectiveness of the PCR protocols and the absence of possible contaminations. Electrophoresis was carried out on 2% agarose gels, prepared using 1× TAE buffer (Tris–acetate–EDTA, pH of 8.3) and stained with Gel Red Nucleic Acid Stain (Biotium Inc., Fremont, CA, USA). PCR products were purified by ExoSAP-IT (USB Corporation, Cleveland, OH, USA) and sequenced for forward and reverse strands (by means of the same primers used for PCR) using an external Sanger sequencing core service (Macrogen Europe, Amsterdam, The Netherlands and Macrogen Europe, Milano, Italy). Noteworthily, dual peaks of similar height, which could be interpreted as evidence of mitochondrial pseudogenes in the nucleus (Numts) or heteroplasmy, were not observed in any of the electropherograms.

### 2.3. Phylogenetic and Phylogeographic Analyses

The 100 newly generated sequences (GenBank #OR782596-OR782695) and the 369 already deposited in GenBank were aligned in their overlapping regions using the package Clustal Omega [70] (available at https://www.ebi.ac.uk/Tools/msa/clustalo/ (accessed on 3 August 2023)).

The genetic variation within the datasets was assessed estimating the number of polymorphic sites (S), the number of haplotypes (H), haplotype diversity (h), and nucleotide diversity (π) using the software package DnaSP 6.12.03 [71].

The best probabilistic model of sequence evolution was determined using jModeltest 2.1.3 [72], with a maximum likelihood optimised search, based on the Akaike (AIC) and Bayesian information criterion (BIC). Both criteria selected the GTR + I + G [73] as the best-fitting model for the dataset.

To infer the genetic relationships among haplotypes and to detect the possible occurrence of discrete genetic clusters, a median-joining network [74] was constructed by means of the software Network 10.2.0.0 (www.fluxus-engineering.com (accessed on 5 September 2023)) (Colchester, UK). Transitions and transversions were equally weighted. Due to the absence of information about the possible appearance of retromutation events, the same weight (10) was assigned to each observed polymorphism.

The principal coordinates analysis (PCoA) was performed using GenAlEX 6.5 [75] on a pairwise p-distance matrix, estimated by using the R packages APE (analysis of phylogenetics and evolution) [76]. This analysis allowed us to identify potential subgroups within the genetic clusters and to determine the dissimilarity represented by the genetic variation among the sequences (see Tran Thi et al. [77]).

Phylogenetic relationships among the Mediterranean *Pinna nobilis* populations and other species belonging to the Pinnidae family were investigated on a dataset including 469 sequences (see Table 1 and Figure 1 for details). Analyses were based on Bayesian inference (BI) and performed by means of the software MrBayes 3.2.7 [78]. The BI was performed by setting as the model parameters the following: NST = 6, rates = invgamma, and ngammacat = 4. Two independent runs consisting each of four Metropolis-coupled MCMC chains (one cold and three heated chains) were run simultaneously for 5,000,000 generations, sampling trees every 1000 generations. The first 25% of the 10,000 sampled trees was then discarded as burn-in (see Scarpa et al. [79]). To assess the convergence of the chains, parameters were verified by using the software Tracer 1.7.1 [80]. In addition, it was checked that the average standard deviation of split frequencies (ASDSFs) approached 0 [78] and the potential scale reduction factor (PSRF) was around 1 [81]. Nodes with a percentage of posterior probability lower than 95% were considered not highly supported. The phylogenetic tree was visualised and edited using FigTree 1.4.0 (http://tree.bio.ed.ac.uk/software/figtree/ (accessed on 9 October 2023)) (see Scarpa et al. [82]).

### 2.4. Estimation of the Divergence Time Analyses

The software package Beast 1.10.4 [83] was used to estimate the divergence time for the clades evidenced by the phylogenetic tree, applying the evolutionary rates proposed by Luttikhuizen et al. [84] for marine bivalves with pelagic larval dispersal. Molecular calibration with fossil data was not applicable in this case, as we aimed to set the molecular clock of the species, and fossil findings cannot trace back to the species level since they only allow for the collocation of the species origin within a temporal range. The mutation rates were set in Beauti (Beast package) by using a normal distribution ranging between 0.14% and 0.52% divergence per nucleotide site per million years. Site parameters were set accordingly to the evolutionary models selected by jModeltest: Substitution Model = GTR; Bases Frequencies = Estimated; Site Heterogeneity Model = Gamma + Invariant Sites; Number of Gamma Categories = 4. For the molecular clock rate variation model, the lognormal uncorrelated relaxed clock was selected, as it assumes independent rates on different branches. For the tree prior, the applied demographic model was the Speciation Yule Process [85,86]. The priors for the model parameters and statistics were determined for calibrating the time-tree assuming the mutation rates per million years. Divergence times were estimated using a normal distribution with lower, central, and upper values set according to the mutation rate per million years. Operator parameters were fixed following the instructions of the user manual. Additionally, the application of the lognormal uncorrelated relaxed clock model provided an indication of how clock-like were the data (measured by the ucld.stdev parameter). If the ucld.stdev parameter estimate was close to 0, then the data were quite clock-like; if the estimated value was much greater than 1, then the data exhibited very substantial rate heterogeneity among the lineages. To obtain the effective sample size (ESS) greater than 200 for all the statistic parameters, a run of 200,000,000 generations was performed, sampling a tree every 20,000 generations following Scarpa et al. [87]. The software Tracer 1.7.1 was also used to view the resulting log file, with the aim of ensuring the convergence of the parameter values to verify whether the ESS values exceeded 200 and to estimate the node ages. Tree Annotator (Beast package) and FigTree were used for drawing, visualising, and editing the time-calibrated tree following Scarpa et al. [87].

## 3. Results

**Figure A1:**
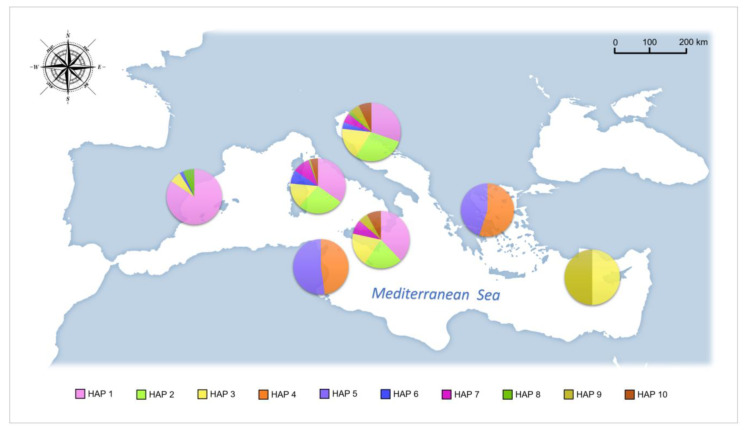
Map showing the distribution of the most common COI haplotypes within the whole Mediterranean basin. In the figure, only haplotypes shared by 5 or more individuals are represented. The number of each haplotype, which is reported in the bottom of the figure, do not refer to a specific nomenclature.

On a total of 469 sequences, a total of 36 polymorphic sites were found, resulting in 49 different haplotypes (see Table 2 and the Figure A1 in Appendix A for the frequency of the distribution of the most common haplotypes in the whole Mediterranean).

The highest levels of genetic variation were found for the populations of the western Mediterranean islands (Corsica, Sardinia, Elba, and Sicily) along with the Adriatic Sea (Venetian Lagoon, Gulf of Trieste, Mljlet, and Telašćica). On the other hand, lower levels of variation were found for the populations from the eastern Mediterranean (the Aegean Sea and Tunisian coastlines). Interestingly, the lowest rates of genetic variation for the whole Mediterranean were found for the Iberian coastlines, even when considering the relevant number of sequences which were analysed.

**Figure 2:**
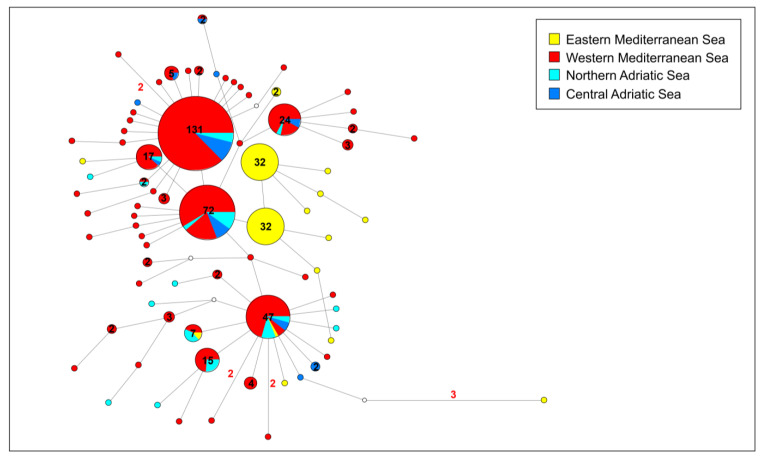
Median-joining network analysis performed on the COI gene fragment. The small white spots on the nodes show median vectors representing the hypothetical sequences that were calculated using the maximum parsimony method. The number of mutations between haplotypes that are greater than n = 1 are reported on the network branches. Additionally, the number of individuals showing the same haplotype that is greater than n = 1 are reported inside the spot.

In the network analysis performed for this dataset (see Figure 2), the sequences were grouped into three different groups according to the genetic Mediterranean structuring proposed for *Pinna nobilis* by Sanna et al. [14]: the western Mediterranean (including sequences from Iberian coastlines, Corsica, Sardinia, Elba Island, and Sicily), the eastern Mediterranean (including sequences from Tunisian coastlines, the Aegean Sea, and Cyprus), and the Adriatic Sea.

Please note that, for the Adriatic group, two similar shades of blue were used to differentiate the network graph sequences from the north of the basin (light blue, including the Venetian Lagoon and Gulf of Trieste) from those belonging to the central part of the basin (sky blue, from the national parks of Mljlet and Telašćica). To test the hypothesis provided by Sanna et al. [14], which set the genetic boundary between the western and eastern Mediterranean for *P. nobilis*, eastward of the Strait of Otranto in the Ionian Sea, Adriatic sequences were divided into the northern and central Adriatic only for the network analysis. Certainly, Sanna et al. [14] proposed that the absence of additional Adriatic populations, aside from the Venetian Lagoon, hinders the confirmation of this hypothesis. In the current study, the inclusion of new sequences from four Adriatic populations in various basin locations enables us to offer insights into the accurate placement of the Mediterranean genetic boundary for this species. Results evidence the occurrence of six highly diffused haplotypes, resulting in at least three main typical network star-like shapes. The three most common haplotypes are diffused in the western Mediterranean and northern and central Adriatic, with the only exception being the third most common of them, which has been also found in one individual from Cyprus. Populations from the Adriatic Sea show a high level of haplotype sharing. These three common haplotypes are surrounded by several diverging haplotypes which, in general, differ for a single point mutation and are private to a single individual. Interestingly, two diverging haplotypes are exclusive to the eastern Mediterranean populations and are shared among Tunisian and Aegean individuals. Interestingly, these haplotypes exhibited divergence from those in the western Mediterranean due to two primary single-nucleotide polymorphisms (SNPs) identified within the last 25 nucleotides of the analysed COI fragment. Specifically, only one of these two distinctive polymorphisms is prevalent in nearly all individuals from both eastern and western populations. This constitutes a silent mutation occurring in the third base of a codon encoding for glycine, involving two purines (transition between the bases A and G). Importantly, this mutation does not induce any amino acid alterations in haplotypes.

The second noteworthy polymorphism, which distinguishes eastern and western populations, is present in a smaller proportion of individuals within western populations. Once again, this represents a silent mutation in the third base of a codon encoding for leucine, involving two pyrimidines (transversion between the bases C and T). Similar to the first polymorphism, this mutation does not result in any amino acid changes in haplotypes.

Overall, eastern Mediterranean populations do not share haplotypes with the western Mediterranean, with the exception of only one haplotype, which was found in seven individuals from Cyprus, Sicily, Venetian Lagoon, and Corsica. The network output evidenced a general high level of genetic variation with a huge number of similar haplotypes differentiating for a few mutations, and with a diffused haplotype sharing both among the populations of the western Mediterranean and the Adriatic, and among the populations of the eastern Mediterranean.

In line with the network analysis, the sequences utilised for the principal coordinates analysis (PCoA) were categorised into three distinct groups: the western Mediterranean, eastern Mediterranean, and Adriatic Sea. The overall findings were in harmony with the network analysis, and the cumulative percentage of variation explained by the first two axes (refer to Supplementary Figure S1 and Supplementary Table S1) just exceeded 50% (axis 1: 34.06%; axis 2: 24.29%). This underscores a general genetic uniformity among the sequences encompassed in the dataset. While the percentage of variation only weakly supported it, the results indicated a genetic structuring between two principal groups of sequences (G1 and G2) along axis 1.

The smaller group (G1), comprising 21.11% of the sequences, predominantly included individuals from the western Mediterranean and the Adriatic Sea, with only two exceptions from the island of Cyprus, two from the Aegean Sea, and two from Tunisian coastlines. In contrast, the larger group (G2) encompassed 78.68% of the sequences and grouped individuals from the western Mediterranean, Adriatic Sea, and eastern Mediterranean. Notably, a single sequence from the Venetian Lagoon (Adriatic Sea) was identified as an outlier, positioned between the two main groups.

**Figure 3:**
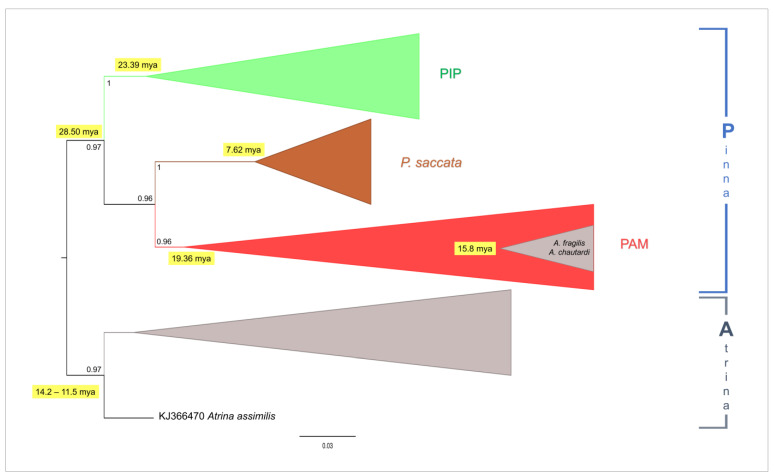
Schematic representation of the Bayesian phylogenetic tree based on the COI gene fragment, which is provided as Figure S2. The values of the node supports are expressed as posterior probabilities.

**Figure 4:**
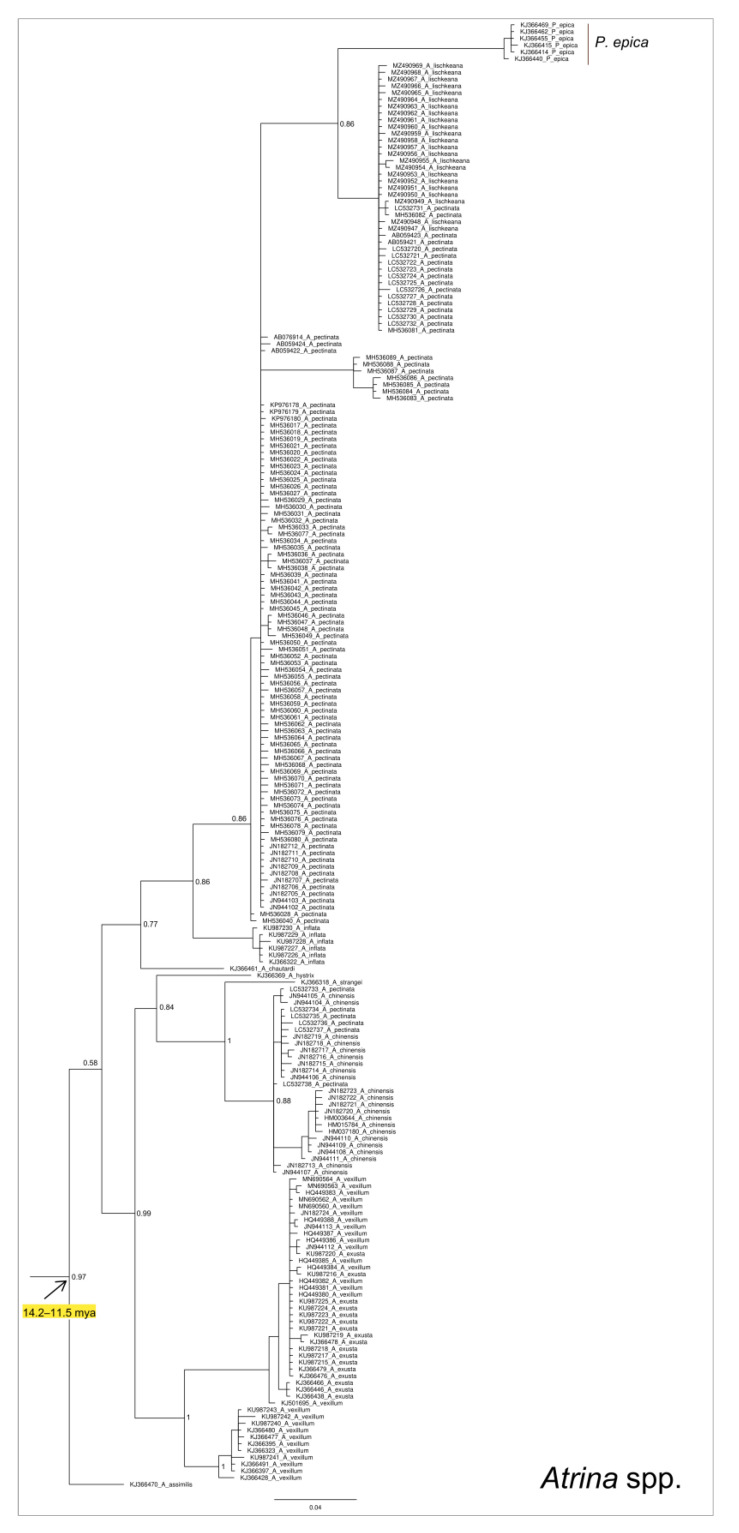
Enlarged detail of the Bayesian phylogenetic tree, which is provided as Supplementary Figure S2, corresponding to the clade A. The values of the node supports are expressed as posterior probabilities.

**Figure 5:**
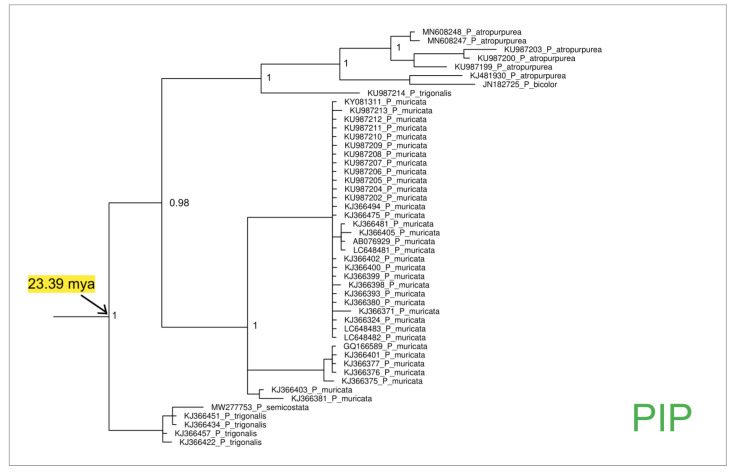
Enlarged detail of the Bayesian phylogenetic tree, which is provided as Supplementary Figure S2, corresponding to the cluster PIP. The values of the node supports are expressed as posterior probabilities.

**Figure 6:**
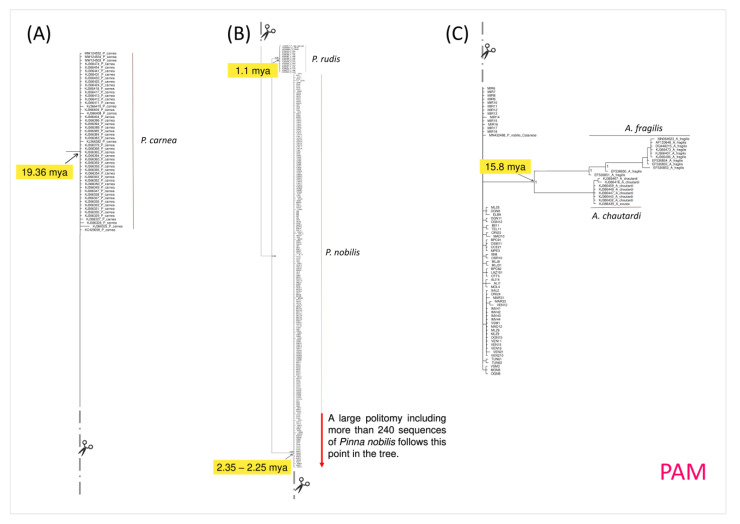
Enlarged details of the Bayesian phylogenetic tree, which is provided as Supplementary Figure S2, corresponding to the cluster PAM. Due to the large size of the PAM cluster, the corresponding figure was here segmented into three parts (**A**–**C**). The section (**A**) represents all sequences of *Pinna carnea* within the dataset; the sections (**B**,**C**) represent sequences of *Pinna rudis* (**B**) and *Pinna nobilis* (**B**,**C**), along with sequences of *Atrina fragilis* and *Atrina chautardi* (**C**). The values of the node supports are expressed as posterior probabilities. Sequences from the present study are the one without the GenBank accession code.

**Figure 7:**
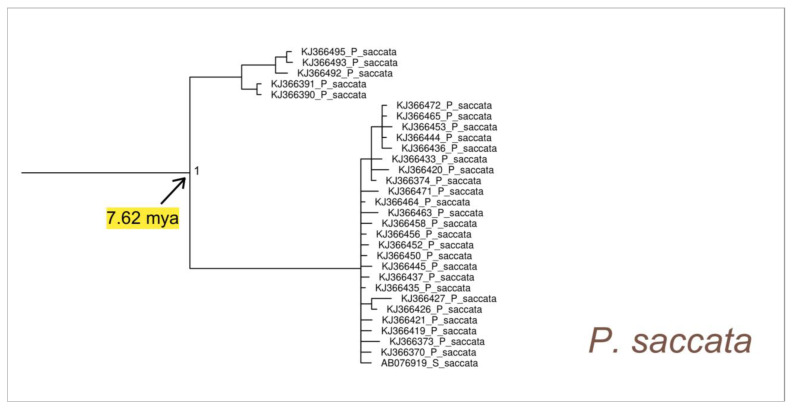
Enlarged detail of the Bayesian phylogenetic tree, which is provided as Supplementary Figure S2, corresponding to the *Pinna saccata* cluster. The values of the node supports are expressed as posterior probabilities.

The Bayesian phylogenetic tree analysis was drawn based on a dataset including not only the *P. nobilis* COI sequences but also the relatives corresponding to all sequences of the species belonging to the family Pinnidae (*Pinna* and *Atrina* genera) available in GenBank so far (see Figure 3 for the schematic representation of the tree, and Figure 4, Figure 5, Figure 6 and Figure 7 and the Supplementary Figure S2 for details on the species and GenBank accession numbers).

Bayesian analyses performed with MrBayes and Beast produced two trees with an identical topology at the main nodes. Accordingly, in the present study, only the tree obtained with MrBayes was presented with the graphical integration of the divergence times data from the ultrametric Beast tree, indicated at the main nodes.

It is noteworthy that, since *Streptopinna* is now considered a junior synonym of *Pinna* [2] based on molecular data, the sequences belonging to this subgenus of *Pinna* were included in the dataset as a species of *Pinna*.

The phylogenetic tree was rooted in the clade representative of the genus *Atrina*, with the aim to specifically infer the relationships among species of the genus *Pinna*. All the main nodes of the tree are well-supported, with values of posterior probabilities (pp) higher than 0.95, with the only exception being the internal large cluster including *Pinna carnea* Gmelin, 1791, *Pinna rudis* Linnaeus, 1758, and *P. nobilis* (pp = 0.55). However, it is important to take into consideration that, within this latter cluster, the nodes of *P. rudis* and *P. nobilis* clades (and the large cluster that includes these two clades) were highly supported.

Results highlight the presence of two monophyletic clades (P and A) representative of the genera *Pinna* (10 species) and *Atrina* (11 species), respectively. The clade A (see Figure 3 and Figure 4 and the Supplementary Figure S2) dates back to a temporal range of 14.2–11.5 (HPD 95%: 3.6–36.64) mya and includes the species belonging to the genus *Atrina*, with the only exception being the *Atrina chautardi* (Nicklès, 1953) and *Atrina fragilis* (Pennant, 1777), which were included in an almost-contemporary (15.84 mya) monophyletic internal subcluster within clade P.

The *Atrina* species clustering within the clade A are almost all diffused in the Indian and Pacific Oceans. However, this clade also includes a well-supported monophyletic cluster, grouping sequences belonging to the species *Pinna epica* Jousseaume, 1894 (see Figure 4 and the Supplementary Figure S2). This discrepancy could be explained considering that this species was recently tentatively designed as *Abyssopinna epica* [88] with the genus *Abyssopinna* Schultz and Huber (2013), classified as a subgenus of *Pinna*. For this reason, the taxonomic status of *P. epica* is still puzzling and deserves to be further investigated from a phylogenetic point of view.

On the other hand, the clade P (see Figure 3 and the Supplementary Figure S2), which represents the genus *Pinna*, dates back to 28.50 (HPD 95%: 16.0–29.0) mya and includes two main clusters (PIP and PAM) (see Figure 3, Figure 4, Figure 5 and Figure 6 and the Supplementary Figure S2) that are representative of Indo-Pacific (PIP) and Atlanto-Mediterranean (PAM) species.

The Indo-Pacific monophyletic cluster PIP (see Figure 3 and Figure 5 and the Supplementary Figure S2) includes species which, in general, are diffused in the Pacific and Indian Oceans and date to 23.39 (HPD 95%: 7.3–69.3) mya.

The Atlanto-Mediterranean cluster PAM (see Figure 3 and Figure 6 and the Supplementary Figure S2) dates back to 19.36 (HPD 95%: 7.4–34.9) mya and includes species from the Atlantic Ocean and Mediterranean Sea.

The sister group of PAM is represented by a well-supported monophyletic cluster that represents the pan-Indo-Pacific species *Pinna saccata* (Linnaeus, 1758) (see Figure 3 and Figure 7 and Figure S2) which dates back to 7.62 (HPD 95%: 1.4–17.5) mya.

Within the cluster PAM (see Figure 6 and the Supplementary Figure S2), the monophyletic polytomic clade of *P. rudis* dates back to 1.1 (HPD 95%: 0.2–3.4) mya, while the large polytomy, which grouped the sequences of *P. nobilis*, dates back to a temporal range of 2.25–2.35 (HPD 95%: 1.29–4.47) mya. This latter cluster of *P. nobilis* is also inclusive of a well-supported monophyletic subcluster grouping the two Atlanto-Mediterranean *Atrina* species, *A. fragilis* and *A. chautardi*.

**Figure 8:**
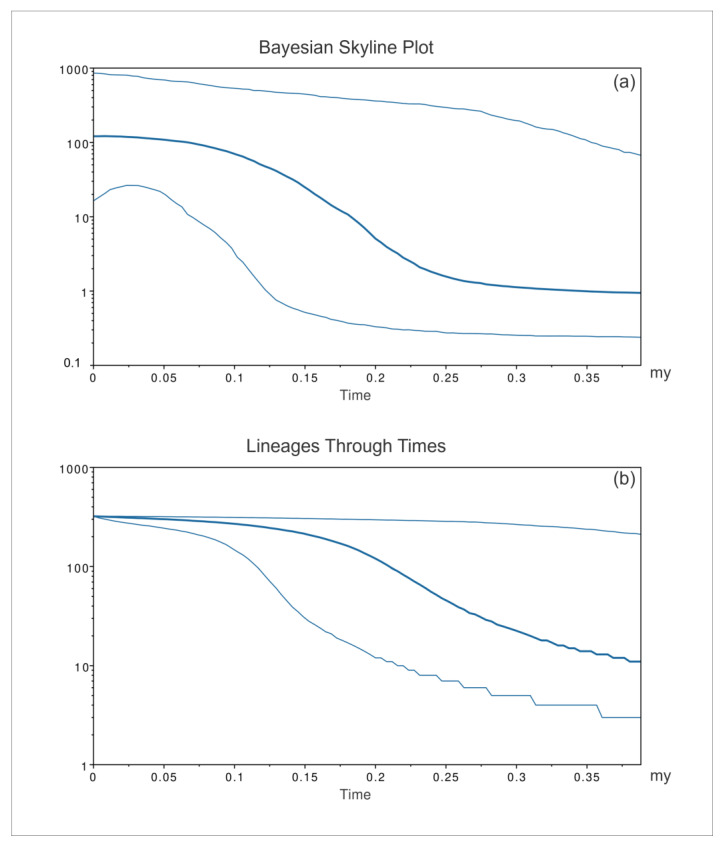
Bayesian skyline plot (**a**) and lineages through time (**b**) for the western Mediterranean populations of *Pinna nobilis*. The effective population size and the number of lineages in the y-axis are shown as a function of million years (my). The thicker central line represents the median value, while the thinnest lines delimit the 95% high posterior density (HPD) region.

**Figure 9:**
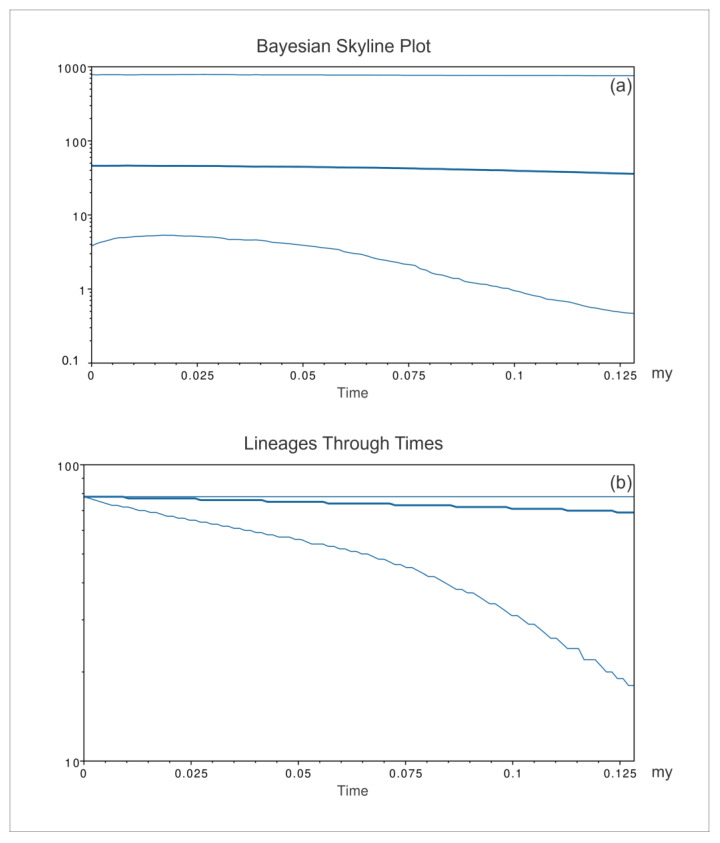
Bayesian skyline plot (**a**) and lineages through time (**b**) for the eastern Mediterranean populations of *Pinna nobilis*. The effective population size and the number of lineages in the y-axis are shown as a function of million years (my). The thicker central line represents the median value, while the thinnest lines delimit the 95% high posterior density (HPD) region.

**Figure 10:**
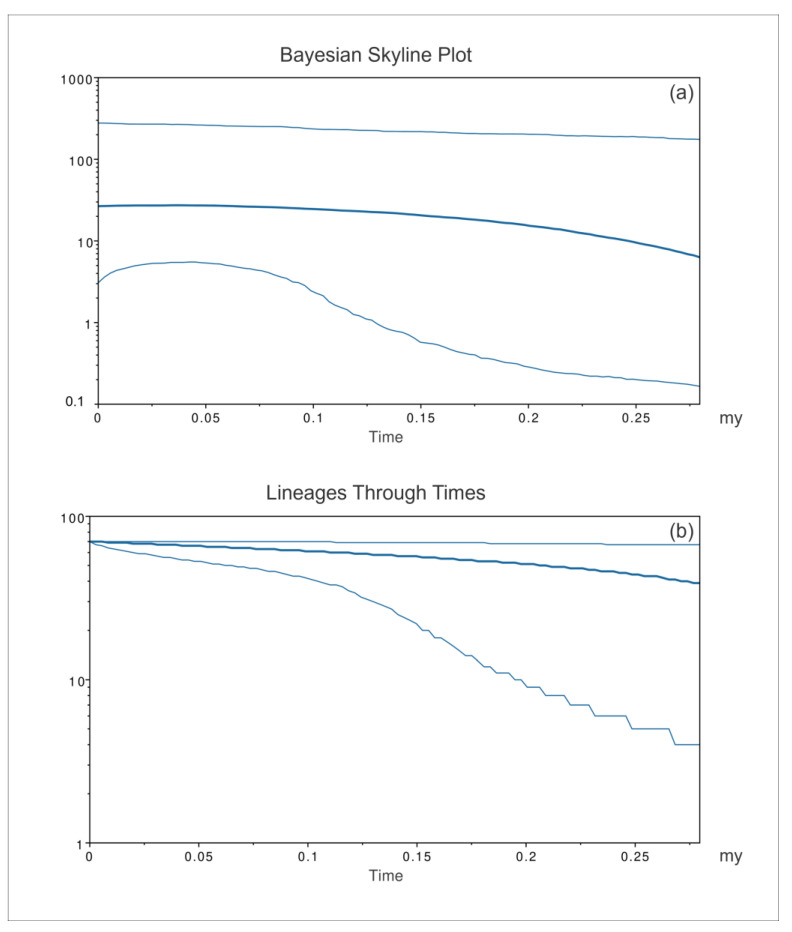
Bayesian skyline plot (**a**) and lineages through time (**b**) for the Adriatic Sea populations of *Pinna nobilis*. The effective population size and the number of lineages in the y-axis are shown as a function of million years (my). The thicker central line represents the median value, while the thinnest lines delimit the 95% high posterior density (HPD) region.

Consistent with the geographic areas considered for network and PCoA analyses, the patterns of *P. nobilis* spread were inferred by the BSP (Figure 8, Figure 9 and Figure 10) for three groups of sequences: the western Mediterranean, eastern Mediterranean, and Adriatic Sea.

The western Mediterranean BSP (*P. nobilis*, Figure 8a) showed an initial long-lasting constant size diffusion of the *P. nobilis* ancestor species from its origin up to 2.5 mya. According to the molecular dating based on the phylogenetic tree analysis, this latter moment (2.5 mya) approximately corresponds to the differentiation of the species *P. nobilis*. The early population of fan mussels experienced a constant exponential expansion of the early population that lasted for about two million years and was followed by a decrease in the population expansion.

Consistently, the analysis also evidenced (Figure 8b) that, from the first radiation of the *P. nobilis* ancestor species, the amount of its mitochondrial COI lineages in the western Mediterranean constantly increased. The species *P. nobilis* differentiated during this exponential growth of mitochondrial lineages (about 2.25–2.35 mya according to the molecular dating of the phylogenetic tree), which lasted for about one further million years after the rise of *P. nobilis*. Starting from this moment, the mitochondrial lineages of *P. nobilis* reduced their growth, reaching a plateau about 0.25 mya.

The eastern Mediterranean BSP (*P. nobilis*, Figure 9a) evidenced a general long-lasting constant size diffusion of *P. nobilis*, which started 1.25 mya. From the first radiation of the species in the eastern Mediterranean, the amount of the mitochondrial COI lineages (Figure 9b) increased, with a constant low level of growth whose extent steadily decreased over time until it reached a plateau approximately 0.25 mya.

The Adriatic Sea BSP (*P. nobilis*, Figure 10a) showed a general trend with a long-lasting constant size expansion of the *P. nobilis* early population, which started approximately 2.5 mya (the early expansion might have been produced by the *P. nobilis* ancestor species). A slight population expansion for *P. nobilis* lasted in the Adriatic Sea for about one million years and was followed by a decrease in the population expansion. The analysis also evidenced that, from the first radiation of *P. nobilis* in the Adriatic, the amount of its mitochondrial COI lineages (Figure 10b) increased, with a general constant low level of growth whose extent steadily decreased over time until it reached a plateau approximately 0.25 mya.

## 4. Discussion

This study, incorporating 21.5% of new *Pinna nobilis* COI sequences from one western Mediterranean and three Adriatic populations not previously investigated, contributes to a more comprehensive understanding of the genetic landscape of *P. nobilis* before the mass mortality event (MME) that pushed the species to the brink of extinction. The choice of the mitochondrial COI marker for the analyses was taken since it was the only one that allowed us to create a dataset of sequences encompassing all the individuals of *P. nobilis* genotyped in the last fifteen years. Importantly, the use of the COI gene enabled the comparison of new molecular data from the present study with populations now extinct, for which it would be impossible to perform analyses with other types of molecular markers.

Consequently, we reconstructed, with the utmost accuracy, the pre-MME phylogeographic patterns of *P. nobilis* in the Mediterranean Sea, shedding light on the origin of the genetic structuring between the western and eastern *P. nobilis* populations hypothesised in Sanna et al. [14] and recently corroborated by Sarafidou et al. [51] based on the analysis of Aegean and Ionian populations surviving after the MME. By utilising samples from populations not yet affected by the mass mortality of *P. nobilis*, we were able to study the evolutionary patterns of the species without the bias of the evolutionary forces that affected the genetic landscape of the populations due to the MME.

The present study infers, with the highest level of resolution possible, the phylogeny of *P. nobilis*, offering, for the first time, insights into the origin of this species. In this context, it must be emphasised that the occurrence of doubly uniparental inheritance (DUI) for mitochondrial DNA, which could impact the reliability of results, has never been reported in *P. nobilis* [14].

### 4.1. Phylogeography and Evolutionary History of *Pinna nobilis*

In accordance with Sanna et al. [14] and Wesselman et al. [58], the present study underscores the presence of a general mitochondrial genetic homogeneity among *Pinna nobilis* populations in the Mediterranean before the MME. Notably, the analysis, incorporating a significantly increased number of new sequences (over 20%), confirms the only exception to this trend reported in Sanna et al. [14], wherein populations from Tunisian coastlines and the Aegean Sea exhibit private haplotypes not found in *P. nobilis* populations from the western Mediterranean sampling sites. However, it should be noted that these exclusive haplotypes from the eastern Mediterranean, which were identified by Katsares et al. [22] and Raboui et al. [25], differ from the western Mediterranean and Adriatic haplotypes by only one or two silent point mutations, which do not impact the amino acid composition of the mitochondrial enzyme cytochrome c oxidase.

Additionally, two sequences from Cyprus showed haplotypes commonly found in the western Mediterranean and Adriatic Sea, and four Tunisian and one Aegean sequence exhibited haplotypes directly derived from those most frequently observed in the western Mediterranean and Adriatic Sea. This suggests that, in the eastern Mediterranean, the prevalent “eastern” haplotypes might have coexisted with several “western” haplotypes that remained undetected due to chance and the limited number of individuals sampled from those areas before the MME. Thus, the entire Mediterranean basin might have been interested by a level of haplotype sharing among western and eastern populations higher than those identified until now. This potential scenario is supported by a recent study on surviving populations from the Aegean and Ionian seas [51], which revealed haplotype sharing between the western Mediterranean and Ionian Sea based on the analysis of a concatenated mitochondrial fragment, including COI and ribosomal 16S genes.

However, it cannot be ruled out that the observed mitochondrial genetic structuring between the western and eastern Mediterranean for *P. nobilis* could result from a selective sweep increasing the frequency of the most adaptive allelic variants in the eastern Mediterranean. The mitochondrial marker used in this study did not reveal any adaptive changes involving protein production for the mutations accounting for the divergence between western and eastern populations. Nevertheless, these haplotypes may be associated with adaptive allelic variants warranting investigation with new nuclear biparental molecular markers in future studies on surviving populations.

The inclusion of new populations from the northern and central Adriatic Sea in this study has provided a better understanding of the level of genetic divergence between this basin and the western Mediterranean. Despite lower genetic variability, our results demonstrate the presence of haplotypes in the Adriatic that also occur in the western Mediterranean. This finding holds significant importance for the LIFE PINNA project (https://www.lifepinna.eu/en/home/), where the transplantation of *P. nobilis* individuals from the northern Adriatic was planned for activities in the western Mediterranean. In light of these results, Adriatic populations of *P. nobilis* have proven to be genetically comparable to those in the western Mediterranean intervention sites (https://www.lifepinna.eu/en/areas-of-intervention/), which have now become extinct.

Furthermore, this study provides a definitive answer to the hypothesis proposed by Sanna et al. [14] regarding the correct position of the genetic boundary spanning the Sicilian Strait. Indeed, this research provides support to the presence of the Mediterranean boundary between western and eastern Mediterranean basins eastward of the Sicilian Strait, in the Ionian Sea.

Several common haplotypes were identified, and their presence and distribution in the network (Figure 2) suggest significant founder effects during the evolutionary history of *P. nobilis*. A similar evolutionary pattern of network star-like shapes has been reported for the mitochondrial COI gene in other species belonging to the family Pinnidae (e.g., *Pinna saccata*, *Pinna muricata*, and *Atrina rigida* (Lightfoot, 1786)) in the Pacific Ocean and the Caribbean Sea [2]. The discovery of this trend in Pinnidae species distributed in vastly different geographic areas suggests a slow mitochondrial mutational substitution rate combined with a high potential for larval dispersal in these species.

The high mitochondrial homogeneity observed in *P. nobilis* is linked to the entry of its ancestor into the Mediterranean from the Atlantic during the Messinian salinity crisis. The Mediterranean became disconnected from the Atlantic Ocean in the late Miocene (5.6 mya). The subsequent Messinian salinity crisis, occurring around this time [68], led to the near-complete desiccation of the basin due to water evaporation. The Zanclean flood event, dated 5.33 mya, marked the return of Atlantic waters to the Mediterranean through the Gibraltar Strait, causing rapid and violent flooding at a rate exceeding ten metres per day [68]. While the return of Atlantic waters might have started weakly and slowly a few thousand years earlier, 90% of the transfer occurred during the Zanclean flood, filling the Mediterranean basin in a short period, ranging from a few months to two years. This event potentially facilitated the early colonisation of the central Mediterranean seabed by larvae belonging to the ancestor of *P. nobilis*, leading to its adaptation and differentiation into the modern, endemic species. This scenario aligns with the detailed description provided by Bianchi et al. [89], which delineates the phases of the Mediterranean Sea refilling post-Zanclean flood along with the migration of Atlantic species into the Mediterranean.

Furthermore, recent identification of *P. nobilis* fossils in a well-studied late Pliocene–early Pleistocene marine succession along the Stirone riverbanks in northern Italy supports this scenario [67]. This region, rich in Pinnidae fossils [90,91,92], likely witnessed the early evolution and differentiation of *P. nobilis*.

Consistent with this proposed scenario, the western Mediterranean populations of *P. nobilis* analysed in this study, covering the coastlines of Corsica, Sardinia, Elba, and Sicily, exhibited the highest levels of genetic variability in the entire Mediterranean. In contrast, the lowest level of genetic variation was observed in *P. nobilis* populations from the Iberian coastlines. This discrepancy may be explained by the direction of the Zanclean flooding, initially involving the central part of the Mediterranean and excluding, at least in the initial phase, the Iberian Peninsula area [93]. The Zanclean flood also created a seabed incision (the Zanclean channel) through the erosion produced by the flooding waters [68,94], establishing a direct connection between the Atlantic and the central Mediterranean through the hydrographic constriction produced by the Strait of Gibraltar [93,95]. The areas laterally distant from this dashing flow were initially excluded [89,93].

The rapid adaptation process of the *P. nobilis* ancestor to Mediterranean conditions, following the settlement of larvae from the Atlantic, might have been facilitated by effective recruitment and fast turnover, as evidenced in the modern fan mussel from the Gulf of Lion [96]. According to the proposed model for the evolution of the Mediterranean Sea level after the Zanclean flood [68], the waters reached the Sicily sill at the end of the first phase of filling, leading the western Mediterranean to attain the maximum marine water level. Only later, did water slowly begin to fill the Adriatic Sea and the eastern Mediterranean through the Sicilian Strait. This sequence of events may explain the overall lower level of *P. nobilis* genetic variation in these two basins.

Our results suggest that *P. nobilis* differentiated from its most recent ancestor in the western Mediterranean approximately 2.3 mya. This estimate aligns with the dating (late Pliocene–early Pleistocene) of *P. nobilis* fossils found in deposits along the Stirone river in northwest Italy [67]. Early populations of the species might have rapidly colonised the Adriatic Sea, where a small number of mitochondrial lineages common in the western Mediterranean are present. Subsequently, the species dispersed in the eastern Mediterranean after more than one million years. This region now features exclusive haplotypes, likely derived from the western Mediterranean, following a typical founder effect model possibly associated to the effect of a selective sweep.

Our study reveals that the diffusion of *P. nobilis* in the Adriatic and eastern Mediterranean, along with its evolutionary rate, advanced slowly, possibly due to biological conditions differentiating these basins from the western Mediterranean (e.g., marine currents, temperature, and salinity), where the species likely spread more rapidly. The species, known to be highly responsive to variations in salinity and temperature [34,50,53,54], and indirectly to the hydrodynamic conditions (which strongly influence the patterns of genetic connectivity for this species, as supposed by González-Wangüemert et al. [59] for modern populations of *P. nobilis*), may have experienced a slower evolutionary rate influenced by climatic changes during the Quaternary glaciations.

Indeed, in the whole Mediterranean, the mitochondrial lineages of *P. nobilis* drastically reduced their growth in the middle Pleistocene, reaching a plateau at the beginning of the Riss glaciation. The severe climatic changes that happened during the Quaternary glaciations might have affected the phylodynamic patterns of *P. nobilis*. For example, during the glacial periods, deeper waters (from below 200 m) flowed into the Mediterranean Sea from the Atlantic [97], while near-surface waters flowed out of the Mediterranean into the Atlantic Ocean across the Straits of Gibraltar [98,99]. In this context, the hydrodynamic of surface waters, where the pelagic larval stage of *P. nobilis* spread, could have been involved in the decreasing of the evolutionary rate of the species.

### 4.2. Phylogeny of *Pinna nobilis*

As previously mentioned, the phylogenetic analysis results indicate that *Pinna nobilis* is an early Pleistocene species, having differentiated approximately 2.3 million years ago in the central Mediterranean after its ancestor entered the region during the Zanclean flood [68]. *Pinna nobilis* is part of a monophyletic cluster that originated in the Miocene period and, in accordance with Lemer et al. [2], includes species from the same genus found in the Atlanto-Mediterranean area, namely, *Pinna carnea* and *Pinna rudis* (see the PAM cluster in the phylogenetic tree shown in Figure 3).

Remarkably, we recognised *P. rudis* as the sister taxon of *P. nobilis*, whose sequences are included within a monophyletic clade that dates to about one million years ago. This finding partially differs from those of Lemer et al. [2], in which *P. rudis* individuals did not form a monophyletic clade and the sequences from Senegal were genetically divergent from those from the Mediterranean, the Azores, and the Canary Islands, and form a sister group to a clade, including the remaining *P. rudis*, *P. nobilis*, and *P. carnea* sequences. Such a discrepancy could be attributable to the increased number of *P. rudis* sequences from the Mediterranean that were included in the present study, as well as to both the molecular marker and the bioinformatic approach used for constructing the phylogenetic tree. Indeed, regarding the latter issue, the phylogenetic tree proposed by Lemer et al. [2] is based on a concatenated heterogeneous dataset of nuclear and mitochondrial regions (18S rRNA, 28S rRNA, 16S rRNA, and COI) that may have led to a scenario somewhat different to that based on our analyses.

It is noteworthy that, in the current investigation, the clade encompassing *P. rudis* underwent differentiation during the Pleistocene epoch (approximately 1 million years ago), coinciding with a decline in the evolutionary rate of *P. nobilis*. This finding can be explained by the fact that, after a founder-flush effect of speciation, colonisation, and adaptation to the habitat of *P. nobilis* in the Mediterranean, the population then stabilised, and *P. rudis*, which evolved more recently, covered some ecological niches less conducive to the development of *P. nobilis*. Specifically, *P. nobilis* favours coastal areas, predominantly seagrass meadows, at depths ranging from 0.5 to 60 m, as well as rocky bottoms or rhodolith beds. In contrast, *P. rudis* displays a preference for small patches of sand in rocky bottoms and rock crevices. While this species can inhabit depths from the surface to 70 m, it is more commonly found at depths between 20 and 70 m, particularly in areas protected from strong water movements, where *P. nobilis* is generally less abundant due to its susceptibility to currents and fronts [100].

In the present scenario, it is interesting to observe that some ecological niches left vacant by *P. nobilis* following mass mortality are now being partially occupied by *P. rudis*. Kersting and Ballesteros [101] documented a behavioural shift in *P. rudis* populations in the Columbretes Islands Marine Reserve off the Iberian Mediterranean coast. Their study revealed a significant increase in the recruitment rates for *P. rudis* after the mass mortality event of *P. nobilis* in 2017. The proposed hypothesis suggests that the local extinction of *P. nobilis* created an opportunity for *P. rudis* to expand, potentially due to the reduced interspecific competition. Given the rarity of high concentrations of both species in the Mediterranean region [102], this occurrence may signify an ongoing process of *P. nobilis* replacement by *P. rudis*.

Moreover, considering the findings of Coupé et al. [47] that some *P. nobilis* individuals that introgressed with *P. rudis* were resistant to infection by *Haplosporidium pinnae*, the expansion of *P. rudis* in the Mediterranean could lead to an increased frequency of hybridisation and introgression with *P. nobilis*. This, in turn, could confer resistance to *H. pinnae*, a primary etiological agent of mass mortality events, thereby potentially promoting the recovery and survival of the fan mussel.

Our findings align with those of Lemer et al. [2], providing support for the notion that the sister group of the cluster encompassing the Atlanto-Mediterranean species of the genus *Pinna* (see the PAM cluster in the phylogenetic tree of Figure 3) comprises an ancient group containing sequences attributed to *Pinna saccata* originating in the early Pliocene. Originally designated as *Streptopinna*, this species underwent recent revision by Lemer et al. [2], classifying *Streptopinna* as a subgenus (status nov.) of *Pinna* and reinstating *P. saccata* as the type species. Our results further affirm the taxonomic reassignment of *Streptopinna saccata* to its new status as an ancient and divergent species within the genus *Pinna*.

The discovery that *P. saccata*, with a pan-Indo-Pacific distribution, is closely related to other *Pinna* species distributed in the Atlanto-Mediterranean region should not be surprising. It is essential to consider that this species traces back to approximately eight million years ago, when the Pacific and Atlantic were still interconnected at the Isthmus of Panama. Indeed, the complete emergence of this land bridge occurred about 4 million years later (3.1–3.5 million years ago), leading to the total isolation between the Caribbean Sea and the Pacific Ocean [79,103,104,105,106].

Interestingly, in addition to *P. saccata*, the species belonging to the genus *Pinna* are divided into two large genetic clusters (PIP and PAM clusters of the phylogenetic tree in Figure 3) on the basis of their geographic origin, the Indo-Pacific and Atlanto-Mediterranean areas, respectively. Having originated in the early Miocene (about 19–23 mya), these clusters are almost contemporary. The beginning of the separation between the Indo-Pacific and the Atlanto-Mediterranean *Pinna* species could be ascribed to the Oligocene adaptive radiation, which gave rise to species very similar to the modern ones. This process might have concluded when the Mediterranean evolved into its current enclosed nature, mostly during Miocene [107], with the closure of the eastern Tethys Sea as Africa and Eurasia (20 mya) [108], and the uplift of the Isthmus of Panama about 3.1–3.5 mya [109,110] that produced the separation of the Atlantic and the Indo-Pacific Oceans.

Overall, our results suggest that the modern genus *Pinna* would have diversified in the Cenozoic era (in the late Oligocene period), which is set earlier than what was previously supposed based on fossils by either Gomez-Alba [3], which fixed its origin in the Miocene period of the Cenozoic era, and Rosewater [111], which fixed its origin in the Jurassic period of the Mesozoic era. This trend has also been observed to be overlapping to that of the genus *Atrina*, since our findings suggest that this taxon is about 15 million years younger than the genus *Pinna*, having originated during the Miocene period of the Cenozoic era. The discrepancy with the previous estimates of its origin based on fossils is relevant because the genus *Atrina* was previously believed to have appeared during the Carboniferous period of the Mesozoic era Rosewater [111].

It is noteworthy that, in our phylogenetic tree, two Atlanto-Mediterranean species of *Atrina* (*Atrina fragilis* and *Atrina chautardi*) do not fall within the clade encompassing all the species of this genus. Instead, they are nested within the clade of *P. nobilis* in a monophyletic cluster that is contemporary to the clade of *Atrina* (see the clade A in the phylogenetic tree in Figure 3). It is crucial to consider that, among *Atrina* species, *A. fragilis* and *A. chautardi* are those predominantly distributed in the Atlantic–Mediterranean region. However, an alternative scenario could be considered to explain the presence of the monophyletic cluster of these two Atlanto-Mediterranean *Atrina* species within the *P. nobilis* clade. This cluster of species, which differentiated in the Miocene, may be representative of the group of ancestor species of *P. nobilis* that originated in the Atlantic Ocean and subsequently migrated into the Mediterranean following the Messinian crisis with the Zanclean flood.

Interestingly, the monophyly of the cluster including *A. fragilis* and *A. chautardi* is partially consistent with Lemer et al. [2], whose maximum clade credibility simplified tree, based on the analysis of concatenated nuclear and mitochondrial genetic data, set the monophyletic cluster of *A. fragilis* and *A. chautardi* in an external position within the clade of *Atrina*.

## 5. Conclusions

The comprehensive reconstruction of the evolutionary history of *Pinna nobilis* presented in this study stands as the most thorough and detailed depiction of phylogeographical and evolutionary patterns available for populations inhabiting distinct basins of the Mediterranean Sea—specifically, the western and eastern Mediterranean, as well as the Adriatic and Aegean seas—prior to the mass mortality event (MME). To attain this objective, we opted to employ the mitochondrial COI gene as the molecular marker, a choice driven by its capacity to facilitate a broad geographic evolutionary investigation, incorporating molecular data from all the populations studied over the past 15 years. The utilisation of the COI gene played a pivotal role in achieving the remarkable degree of accuracy observed in the present study, a precision that would have been challenging to attain without its inclusion.

The obtained results offer novel insights into the evolutionary history of *P. nobilis* populations preceding the MME. These data serve as the foundational basis for (1) appropriately managing survivor individuals/populations and (2) devising effective restocking/transplantation plans in regions where *P. nobilis* faced extinction, as outlined in the action of the European LIFE PINNA project. Particularly concerning the latter point, understanding the evolutionary dynamics of *P. nobilis* and its distribution patterns in the Mediterranean before the catastrophic mortality event holds paramount significance. This knowledge is crucial for repopulation plans, emphasising the need to restore populations with genetic variation as closely aligned as possible to extinct populations. This approach aims to mitigate deleterious genetic drift phenomena that could lead to maladaptation in the newly established populations [112,113,114]. In this context, having a comprehensive understanding of the historical genetic variation and evolutionary trajectory of *P. nobilis* can aid in clarifying how this species has coped with genetic drift (bottlenecks in primis) over time, thus adapting to habitat changes. Having also achieved, by phylogeographic inferences in the present study, new hints on the origin and diffusion of *P. nobilis* after its early differentiation makes it possible to identify the better funders to be used during the plans of population restocking so to minimize the effect of genetic drift.

From a phylogeographic viewpoint, our analyses on the pre-MME genetic variability of *P. nobilis* led to the hypothesis that the extreme and rapid climate changes occurred during the Messinian salinity crisis and the spread of new species into the Mediterranean basin from the Atlantic through the Zanclean channel [68,93,95] could be involved in the appearance of this Pleistocene species which differentiated in the central Mediterranean and shaped the genetic variation observed in its modern populations before the MME. Starting from the knowledge of the distribution of *P. nobilis* genetic variability in the past, the present study laid the foundation to shed light on the evolutionary dynamics characterising this species and help to properly address the conservation management of surviving individuals and the restoration plan of extinct populations. In the future, the analysis of surviving populations from the eastern Mediterranean could highlight the occurrence in these refuge areas (where data currently available report a good number of surviving individuals of *P. nobilis*) of never-described haplotypes that were present in the western Mediterranean before the MME. This finding would be suggestive of the past existence of a large pan-Mediterranean population for *P. nobilis* and, if confirmed by further studies, could provide support for the repopulation plans developed in the western Mediterranean for this species. This scenario could facilitate the conservation of this species, whose restored populations would have a high possibility of maintaining a genetic variation near that of the extinct populations. In this context, Sarafidou et al. [51] analysed the genetic variation in the surviving (sampled in 2018–2021) eastern Mediterranean populations of *P. nobilis* from Greek regions not yet investigated. Results were congruent with the studies performed before the MME [14,58] in evidencing the lack of differentiation among the areas within the eastern Mediterranean and the genetic structuring between the western and eastern basins. Interestingly, the “eastern haplotypes” found by Katsares et al. [22] and Rabaoui et al. [25] were not reported in surviving populations, as a possible consequence of the genetic drift-operated MME, but individuals from the Ionian Sea showed haplotypes that were present in past western and eastern populations, and in modern eastern ones.

In such a context, the Ionian Sea, identified as the genetic boundary between the western and eastern Mediterranean (validated by Sanna et al. [14] and supported by the present study), may serve as a potential overlapping “refuge area”. Here, populations with genetic variability spanning the entire Mediterranean could persist. Although there is a lack of genetic information for *P. nobilis* in this geographic region before the mass mortality event, it holds promise as a significant source area for restocking and conservation initiatives. In the future, further genetic analyses focused on Italian surviving populations in the Ionian Sea, if any, have the potential to illuminate the conservation status of *P. nobilis* and provide insights into its capacity for recovery seven years after the onset of the MME.

## Figures and Tables

**Table 1:** The table reports data on the sampling collection. Sampling sites are indicated for the individuals of *Pinna nobilis* collected during the present study. Details on the sampling locations are also provided for the sequences of *P. nobilis* from all over the Mediterranean taken from GenBank. The accession numbers with an asterisk (*) for Tunisian samples represent the cases for which only haplotypes were provided in GenBank. The group from the Iberian coastlines also includes samples from the site of Banyuls-sur-Mer that is situated on the Mediterranean coast in the French region of Languedoc-Roussillon, just north of the border with Spain.

Sample Code	Sampling Year	Sampling Area	Specimens	GenBank Code	Paper
SARDINIA					
OSR	2013	Ossario (Asinara)	12	OR782596–OR782633	Present Study
ASI	2015	Cala di Scombro di Dentro and Cala Reale (Asinara)	38	OR782634–OR782645	
BPC	2010	Baia di Porto Conte	18	JX854788–JX854805	Sanna et al. [14]
POR	2010	Torre del Porticciolo	3	JX854806–JX854808	
LAZ	2010	Lazzareto	2	JX854809–JX854810	
OSM	2010	Ospedale Marino	21	JX854811–JX854831	
MOL	2010	Molara	11	JX854832–JX854842	
CCE	2010	Capo Ceraso	13	JX854843–JX854855	
SAL	2011	Le Saline	5	JX854856–JX854860	
MPE	2010	Monte Petrosu (Sassi piatti and Isola Cava)	4	JX854861–JX854864	
OTT	2011	Porto Ottiolu	5	JX854865–JX854869	
ORI	2011	Oristano	10	JX854870–JX854879	
MAR	2011	Marceddì	5	JX854880–JX854884	
IMV	2011	Isola di Mal di Ventre	4	JX854885–JX854888	
VMS	2011	Villasimius (Capo Caterina)	4	JX854889–JX854892	
CPA	2011	Costa Paradiso	5	JX854893–JX854897	
MAD	2011	Isola di La Maddalena (Cala Camiciotto)	18	JX854898–JX854915	
CORSICA					
IPI	2011	Isola Piana	13	JX854916–JX854928	Sanna et al. [14]
CPC	2011	Cala Pesciu Cane	12	JX854929–JX854940	
ELBA ISLAND					
ELB	2011	Capo Enfola	10	JX854992–JX855001	Sanna et al. [14]
SICILY					
SVC	2011	San Vito lo Capo (Secca di Cala Rossa)	7	JX854941–JX854947	Sanna et al. [14]
MON	2011	Mondello	11	JX854948–JX854958	
MLZ	2011	Milazzo	10	JX854959–JX854968	
PAC	2011	Pachino (Capo Passero)	8	JX854969–JX854976	
OGN	2011	Ognina di Siracusa	15	JX854977–JX854991	
ADRIATIC SEA					
VEN	2011	Ottagono Alberoni and Santa Maria del Lago	20	JX855002–JX855021	Sanna et al. [14]
MIR	2018	Miramare (Gulf of Trieste)	18	OR782678–OR782695	Present study
TEL	2015	Telašćica–Island Buč	14	OR782646–OR782659	
MLJ	2015	Mljet–Lake Malo Jezero	18	OR782660–OR782677	
CYPRUS					
CYP	2011	Karaoglanoglu	2	JX855022–JX855023	Sanna et al. [14]
AEGEAN SEA					
EPA–EPT	2006-2007	Epanomi	9	DQ448215–DQ448217 EF536827–EF536832	Katsares et al. [22]
AGG	2007	Aggelochori	9	EF536833–EF536841	
XIO	2007	Xios Island	5	EF536842–EF536846	
KOR	2007	Korinthiakos Gulf	3	EF536847–EF536849	
TUNISIAN COASTLINES					
N	2010	Bizerta Lagoon	7	HM998857–HM998866 *	Rabaoui et al. [25]
M	2010	Monastir (Stah Jaber)	9		
S	2010	Kerkennah Island	7		
B	2010	El Bibane Lagoon	9		
K	2010	El Ketef	17		
BIZ	2013	Bizerta Lagoon	1	KF612603	Sanna et al. [57]
IBERIAN COASTLINES					
BAN	2014	Banyuls (France)	9	KY321755–KY321811	Wesselmann et al. [58]
EBR	2014	Ebro Delta (Spain)	9		
IBI	2011	Ibiza (Spain)	10		
MUR	2014	Murcia (Spain)	9		
MALL	2011	Mallorca (Spain)	10		
ALI	2014	Alicante (Spain)	10		

**Table 2:** Sample sizes and genetic diversity estimates obtained for the mitochondrial region analysed for *Pinna nobilis* individuals. N: sample size; S: number of polymorphic sites; H: number of haplotypes; *h*: haplotype diversity; *π*: nucleotide diversity. Populations are labelled as in Table 1. Sites with gaps were not considered.

Sample	N	S	H	*h*	*π*
OSR	12	7	6	0.848	0.005
ASI	38	7	6	0.691	0.004
BPC	18	10	7	0.725	0.005
POR	3	3	2	0.667	0.006
LAZ	2	4	2	1.000	0.012
OSM	21	9	8	0.829	0.006
MOL	11	6	6	0.873	0.006
CCE	13	5	5	0.705	0.005
SAL	5	3	3	0.700	0.003
MPE	4	5	3	0.833	0.007
OTT	5	6	4	0.900	0.008
ORI	10	8	7	0.911	0.007
MAR	5	7	4	0.900	0.009
IMV	4	0	1	0.000	0.000
VMS	4	5	4	1.000	0.008
CPA	5	4	3	0.700	0.005
MAD	18	11	10	0.895	0.007
**Sardinia**	**178**	**28**	**31**	**0.830**	**0.006**
IPI	13	12	9	0.949	0.009
CPC	12	7	6	0.803	0.005
**Corsica**	**25**	**13**	**11**	**0.890**	**0.007**
**Elba Island—ELB**	**10**	**7**	**6**	**0.889**	**0.008**
SVC	7	5	4	0.714	0.005
MON	11	6	6	0.836	0.007
MLZ	10	6	5	0.867	0.007
PAC	8	8	7	0.964	0.007
OGN	15	7	9	0.886	0.007
**Sicily**	**51**	**13**	**16**	**0.882**	**0.007**
VEN	20	10	10	0.895	0.006
MIR	18	5	7	0.791	0.004
TEL	14	9	8	0.890	0.006
MLJ	18	5	5	0.752	0.005
**Adriatic Sea**	**70**	**16**	**21**	**0.870**	**0.007**
**Cyprus—CYP**	**2**	**1**	**2**	**1.000**	**0.003**
EP	9	9	6	0.833	0.007
AG	9	2	3	0.667	0.002
XI	5	2	3	0.700	0.002
KO	3	0	1	0.000	0.000
**Aegean Sea**	**26**	**11**	**8**	**0.720**	**0.004**
N	7	2	3	0.667	0.002
BIZ	1	0	0	0.000	0.000
M	9	4	4	0.694	0.004
S	7	2	3	0.667	0.002
B	9	1	2	0.556	0.002
K	17	1	2	0.382	0.001
**Tunisian coastlines**	**50**	**7**	**7**	**0.621**	**0.003**
BAN	9	3	4	0.750	0.004
EBR	9	2	3	0.417	0.002
IBI	10	3	4	0.533	0.002
MUR	9	0	1	0	0
MALL	10	4	4	0.533	0.003
ALI	10	9	7	0.867	0.010
**Iberian coastlines**	**57**	**15**	**15**	**0.555**	**0.004**
**Whole dataset**	**469**	**36**	**49**	**0.631**	**0.005**

## Data Availability

Sequences obtained in the present study for the mitochondrial cytochrome c oxidase subunit I gene isolated in *Pinna nobilis* from Italy and Croatia were deposited in the GenBank database under the accession numbers OR782596–OR782695.

## Supplementary Materials

The following supporting information can be downloaded at: https://www.mdpi.com/article/10.3390/ani14010114/s1, Figure S1: Principal coordinates analysis performed on the COI gene fragment. Bidimensional plot shows the genetic differentiation among specimens due to the nucleotide substitutions per site found in the dataset. Figure S2: Bayesian phylogenetic tree based on the COI gene fragment analysed in the present study. The values of the node supports are expressed as posterior probabilities. Sequences from the present study are the one without the GenBank accession code. Table S1: Principal coordinates analysis. The table reports the results of the principal coordinates analysis performed on the whole COI fragment dataset.
