# Supplementary material for: Reconstructing the Evolutionary History of Pinna nobilis: New Genetic Signals from the Past of a Species on the Brink of Extinction
Source: Animals (Basel). 2023 Dec 28;14(1):114. doi: 10.3390/ani14010114 (PMC10778441; doi:10.3390/ani14010114)
Supplement: Supplementary file 1 [file animals-14-00114-s001.zip › Table S1.pdf]

Table S1. Principal coordinates analysis. The table reports the results of the principal coordinates analysis performed on the whole COI fragment dataset.

	G1	
**Sample code**	**Sampling site**	**Sampling Area**
MAR33	Marceddì	Sardinia
VEN12	Ottagono Alberoni and Santa Maria del Lago	Venetian Lagoon
OGN8	Ognina di Siracusa	Sicily
OGN9	Ognina di Siracusa	Sicily
MON8	Mondello	Sicily
ELB9	Capo Enfola	Elba Island
VSM3	Villasimius	Sardinia
MLZ6	Milazzo	Sicily
MON3	Mondello	Sicily
MON6	Mondello	Sicily
MON7	Mondello	Sicily
MON11	Mondello	Sicily
IMV44	Isola di Mal di Ventre	Sardinia
MLZ4	Milazzo	Sicily
MLZ8	Milazzo	Sicily
MLZ9	Milazzo	Sicily
MLZ7	Milazzo	Sicily
PAC5	Pachino (Capo Passero)	Sicily
PAC7	Pachino (Capo Passero)	Sicily
SVC6	San Vito lo Capo (Secca di Cala Rossa)	Sicily
IMV42	Isola di Mal di Ventre	Sardinia
VEN21	Ottagono Alberoni and Santa Maria del Lago	Venetian Lagoon
MAD12	Isola di La Maddalena (Cala Camiciotto)	Sardinia
ALI11	Alicante	Spain
ORI11	Oristano	Sardinia
ORI25	Oristano	Sardinia
IPI1	Isola Piana	Corsica
IPI2	Isola Piana	Corsica
MAD13	Isola di La Maddalena (Cala Camiciotto)	Sardinia
MAD15	Isola di La Maddalena (Cala Camiciotto)	Sardinia
ORI24	Oristano	Sardinia
VEN11	Ottagono Alberoni and Santa Maria del Lago	Venetian Lagoon
VEN15	Ottagono Alberoni and Santa Maria del Lago	Venetian Lagoon
VEN16	Ottagono Alberoni and Santa Maria del Lago	Venetian Lagoon
VEN210	Ottagono Alberoni and Santa Maria del Lago	Venetian Lagoon
OTT5	Porto Ottiolu	Sardinia
OSM11	Ospedale Marino	Sardinia
OSM12	Ospedale Marino	Sardinia
OSM14	Ospedale Marino	Sardinia
OSM18	Ospedale Marino	Sardinia
OSM24	Ospedale Marino	Sardinia
OSM25	Ospedale Marino	Sardinia
OTT1	Porto Ottiolu	Sardinia
IBI12	Ibiza	Spain
VEN13	Ottagono Alberoni and Santa Maria del Lago	Venetian Lagoon
VEN17	Ottagono Alberoni and Santa Maria del Lago	Venetian Lagoon
VEN28	Ottagono Alberoni and Santa Maria del Lago	Venetian Lagoon
VEN29	Ottagono Alberoni and Santa Maria del Lago	Venetian Lagoon
VEN110	Ottagono Alberoni and Santa Maria del Lago	Venetian Lagoon
VEN22	Ottagono Alberoni and Santa Maria del Lago	Venetian Lagoon
VEN23	Ottagono Alberoni and Santa Maria del Lago	Venetian Lagoon
VEN26	Ottagono Alberoni and Santa Maria del Lago	Venetian Lagoon
VEN19	Ottagono Alberoni and Santa Maria del Lago	Venetian Lagoon

BIZ1	Bizerta Lagoon	Tunisia
CYP1	Karaoglanoglu	Cyprus
CYP2	Karaoglanoglu	Cyprus
SAL1	Le Saline	Sardinia
SAL2	Le Saline	Sardinia
SAL3	Le Saline	Sardinia
SAL5	Le Saline	Sardinia
OGN2	Ognina di Siracusa	Sicily
OGN10	Ognina di Siracusa	Sicily
OGN15	Ognina di Siracusa	Sicily
MAR31	Marceddì	Sardinia
MAR34	Marceddì	Sardinia
MAR35	Marceddì	Sardinia
CCE4	Capo Ceraso	Sardinia
CCE21	Capo Ceraso	Sardinia
CCE26	Capo Ceraso	Sardinia
MIR5	Miramare (Gulf of Trieste)	Friuli-Venezia Giulia
MIR8	Miramare (Gulf of Trieste)	Friuli-Venezia Giulia
MIR15	Miramare (Gulf of Trieste)	Friuli-Venezia Giulia
IMV41	Isola di Mal di Ventre	Sardinia
IMV43	Isola di Mal di Ventre	Sardinia
TEL5	Telašćica - Island Buč	Croatia
MALL4	Mallorca	Spain
EPT2	Epanomi	Aegean Sea
EPA4	Epanomi	Aegean Sea
MAD7	Isola di La Maddalena (Cala Camiciotto)	Sardinia
BPC91	Baia di Porto Conte	Sardinia
BPC102	Baia di Porto Conte	Sardinia
CPC1	Cala Pesciu Cane	Corsica
CPC7	Cala Pesciu Cane	Corsica
VSM2	Villasimius	Sardinia
VSM1	Villasimius	Sardinia
MPE3	Monte Petrosu (Sassi piatti and Isola Cava)	Sardinia
TUN9	Bizerta Lagoon; Monastir; Kerkennah Island; El Bibane Lagoon; El Ketef *	Tunisia
ALI9	Alicante	Spain
OSR6	Ossario	Sardinia
OSR9	Ossario	Sardinia
ASI11	Cala di Scombro di Dentro and Cala Reale	Sardinia
ELB5	Capo Enfola	Sicily
ELB7	Capo Enfola	Sicily
ELB8	Capo Enfola	Sicily
MLJ6	Mljet - Lake Malo Jezero	Croatia
MLJ8	Mljet - Lake Malo Jezero	Croatia
MLJ20	Mljet - Lake Malo Jezero	Croatia
MLJ31	Mljet - Lake Malo Jezero	Croatia
MLJ33	Mljet - Lake Malo Jezero	Croatia
	**G2**	
**Sample code**	**Sampling site**	**Sampling Area**
TUN61	Bizerta Lagoon; Monastir; Kerkennah Island; El Bibane Lagoon; El Ketef *	Tunisia
TUN62	Bizerta Lagoon; Monastir; Kerkennah Island; El Bibane Lagoon; El Ketef *	Tunisia
MON10	Mondello	Sicily
EPA5	Epanomi	Aegean Sea

EPT5	Epanomi	Aegean Sea
MLJ17	Mljet - Lake Malo Jezero	Croatia
IBI11	Ibiza	Spain
TEL11	Telašćica - Island Buč	Croatia
EPT1	Epanomi	Aegean Sea
EPA3	Epanomi	Aegean Sea
EPA6	Epanomi	Aegean Sea
EPA7	Epanomi	Aegean Sea
EPA8	Epanomi	Aegean Sea
AGG1	Aggelochori	Aegean Sea
AGG2	Aggelochori	Aegean Sea
AGG3	Aggelochori	Aegean Sea
AGG4	Aggelochori	Aegean Sea
AGG5	Aggelochori	Aegean Sea
AGG7	Aggelochori	Aegean Sea
AGG8	Aggelochori	Aegean Sea
AGG9	Aggelochori	Aegean Sea
AGG10	Aggelochori	Aegean Sea
XIO1	Xios Island	Aegean Sea
XIO2	Xios Island	Aegean Sea
XIO3	Xios Island	Aegean Sea
XIO4	Xios Island	Aegean Sea
XIO5	Xios Island	Aegean Sea
KOR1	Korinthiakos Gulf	Aegean Sea
KOR2	Korinthiakos Gulf	Aegean Sea
KOR3	Korinthiakos Gulf	Aegean Sea
TUN11	Bizerta Lagoon; Monastir; Kerkennah Island; El Bibane Lagoon; El Ketef *	Tunisia
TUN12	Bizerta Lagoon; Monastir; Kerkennah Island; El Bibane Lagoon; El Ketef *	Tunisia
TUN13	Bizerta Lagoon; Monastir; Kerkennah Island; El Bibane Lagoon; El Ketef *	Tunisia
TUN14	Bizerta Lagoon; Monastir; Kerkennah Island; El Bibane Lagoon; El Ketef *	Tunisia
TUN15	Bizerta Lagoon; Monastir; Kerkennah Island; El Bibane Lagoon; El Ketef *	Tunisia
TUN16	Bizerta Lagoon; Monastir; Kerkennah Island; El Bibane Lagoon; El Ketef *	Tunisia
TUN17	Bizerta Lagoon; Monastir; Kerkennah Island; El Bibane Lagoon; El Ketef *	Tunisia
TUN18	Bizerta Lagoon; Monastir; Kerkennah Island; El Bibane Lagoon; El Ketef *	Tunisia
TUN19	Bizerta Lagoon; Monastir; Kerkennah Island; El Bibane Lagoon; El Ketef *	Tunisia
TUN110	Bizerta Lagoon; Monastir; Kerkennah Island; El Bibane Lagoon; El Ketef *	Tunisia
TUN111	Bizerta Lagoon; Monastir; Kerkennah Island; El Bibane Lagoon; El Ketef *	Tunisia
TUN112	Bizerta Lagoon; Monastir; Kerkennah Island; El Bibane Lagoon; El Ketef *	Tunisia
TUN113	Bizerta Lagoon; Monastir; Kerkennah Island; El Bibane Lagoon; El Ketef *	Tunisia
TUN114	Bizerta Lagoon; Monastir; Kerkennah Island; El Bibane Lagoon; El Ketef *	Tunisia
TUN115	Bizerta Lagoon; Monastir; Kerkennah Island; El Bibane Lagoon; El Ketef *	Tunisia
TUN116	Bizerta Lagoon; Monastir; Kerkennah Island; El Bibane Lagoon; El Ketef *	Tunisia

TUN117	Bizerta Lagoon; Monastir; Kerkennah Island; El Bibane Lagoon; El Ketef *	Tunisia
TUN118	Bizerta Lagoon; Monastir; Kerkennah Island; El Bibane Lagoon; El Ketef *	Tunisia
TUN2	Bizerta Lagoon; Monastir; Kerkennah Island; El Bibane Lagoon; El Ketef *	Tunisia
TUN3	Bizerta Lagoon; Monastir; Kerkennah Island; El Bibane Lagoon; El Ketef *	Tunisia
TUN4	Bizerta Lagoon; Monastir; Kerkennah Island; El Bibane Lagoon; El Ketef *	Tunisia
TUN51	Bizerta Lagoon; Monastir; Kerkennah Island; El Bibane Lagoon; El Ketef *	Tunisia
TUN52	Bizerta Lagoon; Monastir; Kerkennah Island; El Bibane Lagoon; El Ketef *	Tunisia
TUN53	Bizerta Lagoon; Monastir; Kerkennah Island; El Bibane Lagoon; El Ketef *	Tunisia
TUN54	Bizerta Lagoon; Monastir; Kerkennah Island; El Bibane Lagoon; El Ketef *	Tunisia
TUN55	Bizerta Lagoon; Monastir; Kerkennah Island; El Bibane Lagoon; El Ketef *	Tunisia
TUN56	Bizerta Lagoon; Monastir; Kerkennah Island; El Bibane Lagoon; El Ketef *	Tunisia
TUN57	Bizerta Lagoon; Monastir; Kerkennah Island; El Bibane Lagoon; El Ketef *	Tunisia
TUN58	Bizerta Lagoon; Monastir; Kerkennah Island; El Bibane Lagoon; El Ketef *	Tunisia
TUN59	Bizerta Lagoon; Monastir; Kerkennah Island; El Bibane Lagoon; El Ketef *	Tunisia
TUN510	Bizerta Lagoon; Monastir; Kerkennah Island; El Bibane Lagoon; El Ketef *	Tunisia
TUN511	Bizerta Lagoon; Monastir; Kerkennah Island; El Bibane Lagoon; El Ketef *	Tunisia
TUN512	Bizerta Lagoon; Monastir; Kerkennah Island; El Bibane Lagoon; El Ketef *	Tunisia
TUN513	Bizerta Lagoon; Monastir; Kerkennah Island; El Bibane Lagoon; El Ketef *	Tunisia
TUN514	Bizerta Lagoon; Monastir; Kerkennah Island; El Bibane Lagoon; El Ketef *	Tunisia
TUN515	Bizerta Lagoon; Monastir; Kerkennah Island; El Bibane Lagoon; El Ketef *	Tunisia
TUN516	Bizerta Lagoon; Monastir; Kerkennah Island; El Bibane Lagoon; El Ketef *	Tunisia
TUN517	Bizerta Lagoon; Monastir; Kerkennah Island; El Bibane Lagoon; El Ketef *	Tunisia
TUN518	Bizerta Lagoon; Monastir; Kerkennah Island; El Bibane Lagoon; El Ketef *	Tunisia
TUN519	Bizerta Lagoon; Monastir; Kerkennah Island; El Bibane Lagoon; El Ketef *	Tunisia
TUN520	Bizerta Lagoon; Monastir; Kerkennah Island; El Bibane Lagoon; El Ketef *	Tunisia
TUN521	Bizerta Lagoon; Monastir; Kerkennah Island; El Bibane Lagoon; El Ketef *	Tunisia
TUN522	Bizerta Lagoon; Monastir; Kerkennah Island; El Bibane Lagoon; El Ketef *	Tunisia
TUN7	Bizerta Lagoon; Monastir; Kerkennah Island; El Bibane Lagoon; El Ketef *	Tunisia
TUN8	Bizerta Lagoon; Monastir; Kerkennah Island; El Bibane Lagoon; El Ketef *	Tunisia

TUN10	Bizerta Lagoon; Monastir; Kerkennah Island; El Bibane Lagoon; El Ketef *	Tunisia
TEL2	Telašćica - Island Buč - Island Buč	Croatia
TEL3	Telašćica - Island Buč	Croatia
TEL4	Telašćica - Island Buč	Croatia
TEL6	Telašćica - Island Buč	Croatia
TEL7	Telašćica - Island Buč	Croatia
TEL8	Telašćica - Island Buč	Croatia
TEL9	Telašćica - Island Buč	Croatia
TEL12	Telašćica - Island Buč	Croatia
TEL13	Telašćica - Island Buč	Croatia
TEL14	Telašćica - Island Buč	Croatia
MLJ1	Mljet - Lake Malo Jezero	Croatia
MLJ3	Mljet - Lake Malo Jezero	Croatia
MLJ11	Mljet - Lake Malo Jezero	Croatia
MLJ15	Mljet - Lake Malo Jezero	Croatia
MLJ18	Mljet - Lake Malo Jezero	Croatia
MLJ23	Mljet - Lake Malo Jezero	Croatia
MLJ24	Mljet - Lake Malo Jezero	Croatia
MLJ27	Mljet - Lake Malo Jezero	Croatia
MLJ29	Mljet - Lake Malo Jezero	Croatia
MLJ32	Mljet - Lake Malo Jezero	Croatia
MLJ34	Mljet - Lake Malo Jezero	Croatia
MLJ35	Mljet - Lake Malo Jezero	Croatia
VEN18	Ottagono Alberoni and Santa Maria del Lago	Venetian Lagoon
VEN25	Ottagono Alberoni and Santa Maria del Lago	Venetian Lagoon
VEN27	Ottagono Alberoni and Santa Maria del Lago	Venetian Lagoon
OSR1	Ossario	Sardinia
OSR2	Ossario	Sardinia
OSR3	Ossario	Sardinia
OSR4	Ossario	Sardinia
OSR5	Ossario	Sardinia
OSR8	Ossario	Sardinia
OSR10	Ossario	Sardinia
OSR11	Ossario	Sardinia
OSR12	Ossario	Sardinia
ASI1	Cala di Scombro di Dentro and Cala Reale	Sardinia
ASI2	Cala di Scombro di Dentro and Cala Reale	Sardinia
ASI4	Cala di Scombro di Dentro and Cala Reale	Sardinia
ASI5	Cala di Scombro di Dentro and Cala Reale	Sardinia
ASI6	Cala di Scombro di Dentro and Cala Reale	Sardinia
ASI7	Cala di Scombro di Dentro and Cala Reale	Sardinia
ASI8	Cala di Scombro di Dentro and Cala Reale	Sardinia
ASI10	Cala di Scombro di Dentro and Cala Reale	Sardinia
ASI12	Cala di Scombro di Dentro and Cala Reale	Sardinia
ASI13	Cala di Scombro di Dentro and Cala Reale	Sardinia
ASI14	Cala di Scombro di Dentro and Cala Reale	Sardinia
ASI15	Cala di Scombro di Dentro and Cala Reale	Sardinia
ASI16	Cala di Scombro di Dentro and Cala Reale	Sardinia
ASI17	Cala di Scombro di Dentro and Cala Reale	Sardinia
ASI18	Cala di Scombro di Dentro and Cala Reale	Sardinia
ASI19	Cala di Scombro di Dentro and Cala Reale	Sardinia
ASI20	Cala di Scombro di Dentro and Cala Reale	Sardinia
ASI21	Cala di Scombro di Dentro and Cala Reale	Sardinia
ASI22	Cala di Scombro di Dentro and Cala Reale	Sardinia
ASI25	Cala di Scombro di Dentro and Cala Reale	Sardinia
ASI24	Cala di Scombro di Dentro and Cala Reale	Sardinia
ASI23	Cala di Scombro di Dentro and Cala Reale	Sardinia

ASI26	Cala di Scombro di Dentro and Cala Reale	Sardinia
ASI28	Cala di Scombro di Dentro and Cala Reale	Sardinia
ASI29	Cala di Scombro di Dentro and Cala Reale	Sardinia
ASI31	Cala di Scombro di Dentro and Cala Reale	Sardinia
ASI32	Cala di Scombro di Dentro and Cala Reale	Sardinia
ASI33	Cala di Scombro di Dentro and Cala Reale	Sardinia
ASI34	Cala di Scombro di Dentro and Cala Reale	Sardinia
ASI35	Cala di Scombro di Dentro and Cala Reale	Sardinia
ASI36	Cala di Scombro di Dentro and Cala Reale	Sardinia
ASI37	Cala di Scombro di Dentro and Cala Reale	Sardinia
ASI38	Cala di Scombro di Dentro and Cala Reale	Sardinia
BAN5	Banyuls	Spain
BAN16	Banyuls	Spain
BAN1	Banyuls	Spain
EBR18	Ebro Delta	Spain
EBR21	Ebro Delta	Spain
EBR22	Ebro Delta	Spain
IBI4	Ibiza	Spain
IBI5	Ibiza	Spain
IBI6	Ibiza	Spain
IBI7	Ibiza	Spain
IBI8	Ibiza	Spain
IBI9	Ibiza	Spain
IBI10	Ibiza	Spain
MUR3	Murcia	Spain
MUR7	Murcia	Spain
MUR15	Murcia	Spain
MUR19	Murcia	Spain
MALL3	Mallorca	Spain
MALL5	Mallorca	Spain
MALL6	Mallorca	Spain
MALL8	Mallorca	Spain
ALI5	Alicante	Spain
BAN21	Banyuls	Spain
EBR8	Ebro Delta	Spain
EBR13	Ebro Delta	Spain
EBR19	Ebro Delta	Spain
IBI3	Ibiza	Spain
MALL1	Mallorca	Spain
MALL7	Mallorca	Spain
MALL9	Mallorca	Spain
ALI13	Alicante	Spain
ALI16	Alicante	Spain
ALI4	Alicante	Spain
EBR4	Ebro Delta	Spain
MUR10	Murcia	Spain
BAN14	Banyuls	Spain
BAN15	Banyuls	Spain
BAN19	Banyuls	Spain
ALI8	Alicante	Spain
ALI3	Alicante	Spain
EBR5	Ebro Delta	Spain
EBR6	Ebro Delta	Spain
MUR13	Murcia	Spain
MALL10	Mallorca	Spain
MALL2	Mallorca	Spain
BAN3	Banyuls	Spain
BAN4	Banyuls	Spain

MUR11	Murcia	Spain
MUR14	Murcia	Spain
MUR16	Murcia	Spain
BPC1	Baia di Porto Conte	Sardinia
BPC25	Baia di Porto Conte	Sardinia
BPC28	Baia di Porto Conte	Sardinia
BPC38	Baia di Porto Conte	Sardinia
BPC45	Baia di Porto Conte	Sardinia
BPC51	Baia di Porto Conte	Sardinia
BPC52	Baia di Porto Conte	Sardinia
BPC72	Baia di Porto Conte	Sardinia
BPC81	Baia di Porto Conte	Sardinia
BPC101	Baia di Porto Conte	Sardinia
BPC111	Baia di Porto Conte	Sardinia
BPC121	Baia di Porto Conte	Sardinia
BPC122	Baia di Porto Conte	Sardinia
BPC131	Baia di Porto Conte	Sardinia
BPC132	Baia di Porto Conte	Sardinia
POR11	Torre del Porticciolo	Sardinia
POR12	Torre del Porticciolo	Sardinia
LAZ152	Lazzareto	Sardinia
OSM1	Ospedale Marino	Sardinia
OSM3	Ospedale Marino	Sardinia
OSM4	Ospedale Marino	Sardinia
OSM5	Ospedale Marino	Sardinia
OSM6	Ospedale Marino	Sardinia
OSM7	Ospedale Marino	Sardinia
OSM9	Ospedale Marino	Sardinia
OSM13	Ospedale Marino	Sardinia
OSM17	Ospedale Marino	Sardinia
OSM19	Ospedale Marino	Sardinia
OSM22	Ospedale Marino	Sardinia
OSM26	Ospedale Marino	Sardinia
OSM27	Ospedale Marino	Sardinia
MOL1	Molara	Sardinia
MOL3	Molara	Sardinia
MOL6	Molara	Sardinia
MOL7	Molara	Sardinia
MOL8	Molara	Sardinia
MOL12	Molara	Sardinia
CCE1	Capo Ceraso	Sardinia
CCE2	Capo Ceraso	Sardinia
CCE3	Capo Ceraso	Sardinia
CCE5	Capo Ceraso	Sardinia
CCE22	Capo Ceraso	Sardinia
CCE23	Capo Ceraso	Sardinia
CCE24	Capo Ceraso	Sardinia
CCE25	Capo Ceraso	Sardinia
CCE27	Capo Ceraso	Sardinia
CCE212	Capo Ceraso	Sardinia
SAL4	Le Saline	Sardinia
MPE1	Monte Petrosu (Sassi piatti and Isola Cava)	Sardinia
MPE2	Monte Petrosu (Sassi piatti and Isola Cava)	Sardinia
MPE21	Monte Petrosu (Sassi piatti and Isola Cava)	Sardinia
OTT2	Porto Ottiolu	Sardinia
OTT4	Porto Ottiolu	Sardinia
ORI12	Oristano	Sardinia
ORI13	Oristano	Sardinia

ORI14	Oristano	Sardinia
ORI15	Oristano	Sardinia
ORI21	Oristano	Sardinia
ORI22	Oristano	Sardinia
ORI23	Oristano	Sardinia
MAR32	Marceddì	Sardinia
VSM2	Villasimius	Sardinia
VSM4	Villasimius	Sardinia
CPA2	Costa Paradiso	Sardinia
CPA3	Costa Paradiso	Sardinia
CPA4	Costa Paradiso	Sardinia
CPA5	Costa Paradiso	Sardinia
MAD1	Isola di La Maddalena (Cala Camiciotto)	Sardinia
MAD2	Isola di La Maddalena (Cala Camiciotto)	Sardinia
MAD4	Isola di La Maddalena (Cala Camiciotto)	Sardinia
MAD5	Isola di La Maddalena (Cala Camiciotto)	Sardinia
MAD6	Isola di La Maddalena (Cala Camiciotto)	Sardinia
MAD8	Isola di La Maddalena (Cala Camiciotto)	Sardinia
MAD9	Isola di La Maddalena (Cala Camiciotto)	Sardinia
MAD10	Isola di La Maddalena (Cala Camiciotto)	Sardinia
MAD11	Isola di La Maddalena (Cala Camiciotto)	Sardinia
MAD14	Isola di La Maddalena (Cala Camiciotto)	Sardinia
MAD16	Isola di La Maddalena (Cala Camiciotto)	Sardinia
MAD17	Isola di La Maddalena (Cala Camiciotto)	Sardinia
MAD18	Isola di La Maddalena (Cala Camiciotto)	Sardinia
IPI3	Isola Piana	Corsica
IPI4	Isola Piana	Corsica
IPI5	Isola Piana	Corsica
IPI6	Isola Piana	Corsica
IPI8	Isola Piana	Corsica
IPI9	Isola Piana	Corsica
IPI12	Isola Piana	Corsica
IPI14	Isola Piana	Corsica
CPC2	Cala Pesciu Cane	Corsica
CPC4	Cala Pesciu Cane	Corsica
CPC5	Cala Pesciu Cane	Corsica
CPC8	Cala Pesciu Cane	Corsica
CPC9	Cala Pesciu Cane	Corsica
CPC10	Cala Pesciu Cane	Corsica
CPC12	Cala Pesciu Cane	Corsica
CPC13	Cala Pesciu Cane	Corsica
CPC14	Cala Pesciu Cane	Corsica
SVC1	San Vito lo Capo (Secca di Cala Rossa)	Sicily
SVC2	San Vito lo Capo (Secca di Cala Rossa)	Sicily
SVC3	San Vito lo Capo (Secca di Cala Rossa)	Sicily
SVC4	San Vito lo Capo (Secca di Cala Rossa)	Sicily
SVC5	San Vito lo Capo (Secca di Cala Rossa)	Sicily
SVC7	San Vito lo Capo (Secca di Cala Rossa)	Sicily
MON1	Mondello	Sicily
MON2	Mondello	Sicily
MON4	Mondello	Sicily
MON5	Mondello	Sicily
MON9	Mondello	Sicily
MLZ1	Milazzo	Sicily
MLZ2	Milazzo	Sicily
MLZ5	Milazzo	Sicily
PAC1	Pachino (Capo Passero)	Sicily
PAC2	Pachino (Capo Passero)	Sicily

PAC4	Pachino (Capo Passero)	Sicily
PAC6	Pachino (Capo Passero)	Sicily
PAC8	Pachino (Capo Passero)	Sicily
OGN1	Ognina di Siracusa	Sicily
OGN3	Ognina di Siracusa	Sicily
OGN4	Ognina di Siracusa	Sicily
OGN5	Ognina di Siracusa	Sicily
OGN6	Ognina di Siracusa	Sicily
OGN7	Ognina di Siracusa	Sicily
OGN11	Ognina di Siracusa	Sicily
OGN12	Ognina di Siracusa	Sicily
OGN13	Ognina di Siracusa	Sicily
OGN14	Ognina di Siracusa	Sicily
ELB3	Capo Enfola	Sicily
ELB4	Capo Enfola	Sicily
ELB6	Capo Enfola	Sicily
MIR1	Miramare (Gulf of Trieste)	Friuli-Venezia Giulia
MIR2	Miramare (Gulf of Trieste)	Friuli-Venezia Giulia
MIR3	Miramare (Gulf of Trieste)	Friuli-Venezia Giulia
MIR4	Miramare (Gulf of Trieste)	Friuli-Venezia Giulia
MIR6	Miramare (Gulf of Trieste)	Friuli-Venezia Giulia
MIR7	Miramare (Gulf of Trieste)	Friuli-Venezia Giulia
MIR9	Miramare (Gulf of Trieste)	Friuli-Venezia Giulia
MIR10	Miramare (Gulf of Trieste)	Friuli-Venezia Giulia
MIR11	Miramare (Gulf of Trieste)	Friuli-Venezia Giulia
MIR12	Miramare (Gulf of Trieste)	Friuli-Venezia Giulia
MIR13	Miramare (Gulf of Trieste)	Friuli-Venezia Giulia
MIR14	Miramare (Gulf of Trieste)	Friuli-Venezia Giulia
MIR16	Miramare (Gulf of Trieste)	Friuli-Venezia Giulia
MIR17	Miramare (Gulf of Trieste)	Friuli-Venezia Giulia
MIR18	Miramare (Gulf of Trieste)	Friuli-Venezia Giulia
MOL9	Molara	Sardinia
TEL1	Telašćica - Island Buč	Croatia
MLZ3	Milazzo	Sicily
MOL5	Molara	Sardinia
MOL2	Molara	Sardinia
MOL4	Molara	Sardinia
TEL10	Telašćica - Island Buč	Croatia
MOL11	Molara	Sardinia
MLZ10	Milazzo	Sicily
VEN24	Ottagono Alberoni and Santa Maria del Lago	Venetian Lagoon
ELB1	Capo Enfola	Sicily
ELB2	Capo Enfola	Sicily

ELB10	Capo Enfola	Sicily
CPC11	Cala Pesciu Cane	Corsica
BPC82	Baia di Porto Conte	Sardinia
POR13	Torre del Porticciolo	Sardinia
ALI1	Alicante	Spain
ALI14	Alicante	Spain
CPA1	Costa Paradiso	Sardinia
IPI10	Isola Piana	Corsica
IPI11	Isola Piana	Corsica
IPI15	Isola Piana	Corsica
LAZ151	Lazzareto	Sardinia
PAC3	Pachino (Capo Passero)	Sicily
MAD3	Isola di La Maddalena (Cala Camiciotto)	Sardinia
OTT3	Porto Ottiolu	Sardinia
OSM8	Ospedale Marino	Sardinia
ASI3	Cala di Scombro di Dentro and Cala Reale	Sardinia
OSR7	Ossario	Sardinia
ASI9	Cala di Scombro di Dentro and Cala Reale	Sardinia
ASI30	Cala di Scombro di Dentro and Cala Reale	Sardinia
ASI27	Cala di Scombro di Dentro and Cala Reale	Sardinia
