# Supplementary figures and images for: Reconstructing the Evolutionary History of Pinna nobilis: New Genetic Signals from the Past of a Species on the Brink of Extinction
Source: Animals (Basel). 2023 Dec 28;14(1):114. doi: 10.3390/ani14010114 (PMC10778441; doi:10.3390/ani14010114)

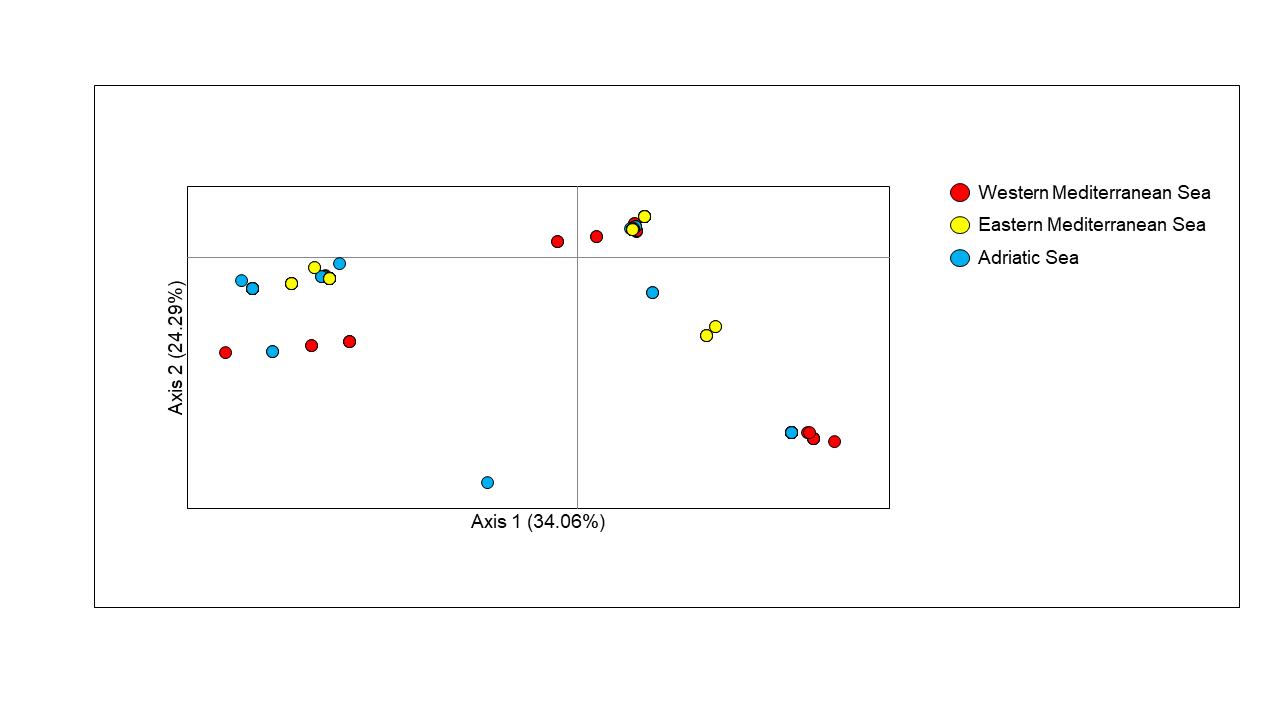

Supplement: Supplementary file 1 [file animals-14-00114-s001.zip › Figure S1.tif]
