# Supplementary material for: Reconstructing the Evolutionary History of Pinna nobilis: New Genetic Signals from the Past of a Species on the Brink of Extinction
Source: Animals (Basel). 2023 Dec 28;14(1):114. doi: 10.3390/ani14010114 (PMC10778441; doi:10.3390/ani14010114)
Supplement: Supplementary file 1 [file Figure S2.pdf › Figure S2.pdf]

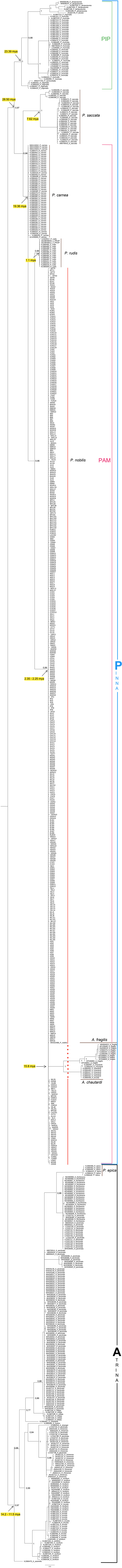

23.39 mya
PIP
26.50 mya
7.02 mya
P. saccata
P. carnea
19.36 mya
1.1 mya
P. rudis
P. nobilis
PAM
2.35 - 2.25 mya
P
I
N
N
A
A. fragilis
A. chautardi
15.8 mya
P. epica
A
T
R
I
N
A
14.2 - 11.5 mya
